# Risk factor screening and predictive modeling of time-in-range in patients with T2DM undergoing SIIT therapy

**DOI:** 10.3389/fendo.2025.1664366

**Published:** 2025-12-04

**Authors:** Yujia Liu, Wanting Liu, Zhifeng Jin, Nengjuan Li, Lingyong Cao, Jiang Hu, Shuyuan Lin

**Affiliations:** 1 College of Basic Medical Sciences, Zhejiang Chinese Medical University, Hangzhou, Zhejiang, China; 2 Department of Endocrinology, The Second Affiliated Hospital of Zhejiang Chinese Medical University, Hangzhou, Zhejiang, China

**Keywords:** type 2 diabetes, TIR, SIIT, retrospective study, real-world

## Abstract

**Objective:**

To identify the risk factors that influence the time in range (TIR) of blood glucose during hospitalization in patients with type 2 diabetes mellitus (T2DM) undergoing short-term intensive insulin therapy (SIIT), and to establish a predictive model for in-hospital blood glucose fluctuations based on real-world data.

**Methods:**

Retrospective data of T2DM patients who were admitted to the Second Affiliated Hospital of Zhejiang Chinese Medicine University for SIIT between 2017 and March 2024 were collected. Random allocation was used to divide the dataset into a training set and a validation set at a ratio of 7:3. Prediction models were constructed separately using logistic regression and random forest algorithms. Additionally, a nomogram was developed for facilitating clinical application.

**Results:**

A total of 796 T2DM patients who received SIIT were included, with 651 achieving TIR ≥ 70% within 10 days of hospitalization. Increasing age, fasting blood glucose (FBG), and use of glinides had a negative effect on achieving TIR ≥ 70%. In contrast, female sex and higher lymphocyte count were associated with increased likelihood of achieving TIR ≥ 70%. In the subgroup analysis, FBG, the presence of diabetic nephropathy (DN), and the occurrence of major adverse cardiovascular events (MACE) were found to potentially reduce the risk of achieving both TIR ≥ 70% and TITR ≥ 50% within 10 days of hospitalization. For model performance evaluation, the logistic regression model demonstrated slightly superior predictive accuracy (F1 score = 0.89, AUC = 0.80) compared with the random forest model (F1 score = 0.84, AUC = 0.72) on the full sample. After applying undersampling, the model’s ability to correctly identify negative cases improved, with specificity increasing to 0.53.

**Conclusion:**

This study, based on real-world data, developed a machine learning model (including logistic regression and random forest) to predict the achievement of TIR during hospitalization. The model not only identifies key clinical factors influencing blood glucose fluctuations, but also provides quantifiable decision support for personalized glucose management. This model has the potential to offer new insights and methods for early identification of high-risk patients and optimization of SIIT treatment strategies in clinical practice.

## 1 Introduction

T2DM poses a significant global health challenge, with its prevalence rising substantially over the past few decades, currently affecting more than 537 million people worldwide. China has the highest number of individuals with diabetes, with approximately 12.4% of adults impacted by this condition (1). Despite the variety of available anti-diabetic medications, about 50% of patients in China still do not achieve adequate glycemic control (2).

SIIT aims to achieve precise blood glucose control through short-term, frequent insulin administration. This therapeutic approach can effectively and rapidly reduce the blood glucose level while minimizing the risk of acute complications. In the long term, SIIT has been shown to improve retinal microvascular conditions and reduce the incidence of microvascular complications. It also decreases the risk of cardiovascular events, such as myocardial infarction, and lowers all-cause mortality (3). Furthermore, several studies have shown that SIIT can significantly enhance islet β cell function and insulin sensitivity in patients with early-stage T2DM (4–7), potentially reversing the progression of diabetes to some extent. Consequently, the guidelines from the Chinese Diabetes Society recommend SIIT as the standard treatment for newly diagnosed T2DM with significant hyperglycemia (8).

Glycated hemoglobin (HbA1c), a traditional indicator of glycemic fluctuations, reflects the average blood glucose level over the past 2–3 months and has long been regarded as a standard biomarker for assessing blood glucose control in patients with T2DM (9). However, it has limitations in evaluating short-term blood glucose variability, particularly in T2DM patients who are hospitalized for poor blood glucose control and receiving SIIT. In previous studies, fasting blood glucose (FBG) and two-hour postprandial blood glucose (2h-PBG) have often served as targets for T2DM patients receiving SIIT (FBG < 6.1 mmol/L, 2h-PBG < 8.0 mmol/L) (7). However, FBG and 2h-PBG only represent blood glucose control at specific time points and cannot fully capture daily blood glucose fluctuations. In recent years, TIR for blood glucose, along with the derived metrics of time above range (TAR) and time below range (TBR), has been recommended as new indicators for assessing glycemic variability (GV) index and blood glucose control (10). TIR is not only closely related to HbA1c, but also can reflect short-term blood glucose fluctuations. It overcomes some limitations of HbA1c to a certain extent, and can be used as an index to evaluate the blood glucose fluctuations of T2DM patients receiving SIIT. Studies have shown that TIR > 80% in critically ill patients treated with intravenous insulin is associated with lower mortality (11). Additionally, controlling daily blood glucose variability in T2DM patients during SIIT helps improve pancreatic β-cell function and increases the likelihood of achieving long-term remission (12).

Several studies have explored potential factors influencing the achievement of target time in range (TIR). For example, Sun et al. proposed a random forest model based on fasting plasma glucose (FPG) (13), postprandial plasma glucose (PPG), and HbA1c to predict TIR (14). Other researchers have reported a nonlinear relationship between the triglyceride–glucose (TyG) index and TIR. They found that a lower TyG index may be associated with better glycemic control and a higher TIR attainment rate. However, most of these studies have focused on outpatient populations. Research on hospitalized patients receiving short-term intensive insulin therapy (SIIT) is limited. The determinants of TIR attainment in this subset of patients, including baseline glycemic level, pancreatic β-cell function, and the presence of diabetic complications, have not yet been fully elucidated.

Therefore, this study retrospectively collected data from 796 hospitalized T2DM patients receiving SIIT to investigate changes in their TIR during hospitalization. By constructing logistic regression and random forest predictive models, we evaluated the clinical utility of the models from four aspects: accuracy, recall, precision, and specificity, along with the F1 Score. We compared the performance of the two models to identify factors affecting TIR outcomes, with the aim of enhancing clinical understanding of blood glucose variability in SIIT patients and assisting in stable glycemic control and subsequent glucose management.

## 2 Method

### 2.1 Study design

#### 2.1.1 Study population and data sources

We retrospectively collected data from patients with T2DM who underwent SIIT at the Second Affiliated Hospital of Zhejiang Chinese Medical University (Xinhua Hospital of Zhejiang Province) between March 2017 and March 2024. This study was conducted in accordance with the Strengthening the Reporting of Observational Studies in Epidemiology (STROBE) statement and the principles of the Declaration of Helsinki, and was approved by the Ethics Review Committee of the Second Affiliated Hospital of Zhejiang Chinese Medical University (approval number: Lunshen 2023yan No. 089-01). All enrolled patients were exempted from providing informed consent due to the retrospective nature of the study.

#### 2.1.2 Inclusion criteria

1. Primary diagnosis of T2DM (E11.900) or T2DM with poor glycemic control (E11.600x051).
2. Age between 18 and 90 years, gender unlimited.
3. Patients must have a hospitalization duration of 7 days or more, during which they receive SIIT.
4. The number of effective fingertip blood glucose monitoring per day is greater than or equal to 6 times, and the earliest and latest measurement time interval must be more than 12 hours. The number of hospitalization days with effective fingertip blood glucose test must account for more than 80% of the total hospitalization days.
5. The first measurement date of the GV index must be no later than 2 days after admission, the last measurement of the GV index must be no earlier than 3 days before discharge, and the interval between any two consecutive GV index measurements must not exceed 2 days.
6. Patients must have results for HbA1c test conducted within 2 months prior to the admission date, and other laboratory tests are accepted only if conducted within 1 week before admission or within 2 days after admission.
7. For patients who are re-hospitalized during the study period, only the first diagnosed case will be included.

#### 2.1.3 Exclusion criteria

1. Other types of diabetes: type 1 diabetes, gestational diabetes, late-onset autoimmune diabetes in adults, etc.
2. Patients who did not undergo routine blood and lipid tests in the laboratory examination 2 weeks before admission and within 2 days after admission;
3. Patients diagnosed with hypoglycemic coma, hyperglycemic hyperosmolar syndrome coma, diabetic ketoacidosis, and other diabetes mellitus with severe acute complications;
4. Grade III (severe) or above hypertension: systolic blood pressure ≥ 160 mmHg or diastolic blood pressure ≥ 100 mmHg;
5. Patients with severe renal impairment: chronic renal failure azotemia stage, chronic renal insufficiency uremia stage, end-stage renal disease, blood urea nitrogen (BUN) ≥ 100 mg/dl, blood creatinine (SCR) ≥ 5mg/dl, acute renal failure or receiving dialysis treatment;
6. Patients with severe liver function damage: acute viral hepatitis, EB viral hepatitis, drug-induced liver injury, severe alcoholic liver disease, liver failure, hepatic encephalopathy, ALT or AST increase more than 5 times the upper limit of normal, etc;
7. There are acute major cardiovascular events such as acute myocardial infarction, acute stroke, acute heart failure in the diagnosis;
8. Diagnosis of other diseases that affect blood glucose control, such as: acute and chronic pancreatitis, pancreatic cancer, pancreatectomy and other pancreatic diseases; Cushing’s syndrome (Cushing’s Syndrome), acromegaly (Acromegaly) and other endocrine diseases; or fungal infection of the urinary tract, Candida vaginitis, urinary tract infection, herpes zoster, syphilis and other infections and stress; autoimmune diseases such as Graves’ disease;
9. Diagnosed with malignant tumors such as prostate malignant tumor, lung malignant tumor, Kaposi’s sarcoma, etc., or pituitary adenoma, parathyroid tumor, adrenal adenoma, pheochromocytoma, etc., which cause direct glucose or insulin;
10. Excluding the uncertainty of Chinese medicine treatment such as inability to determine the dose of Chinese medicine and the method of use.

### 2.2 Data processing

#### 2.2.1 Data collection

The electronic medical record data, including diagnoses, medical orders, laboratory tests, and fingertip blood glucose records, were directly exported from the hospital’s scientific research management system. A standardized spreadsheet was designed to organize the retrospective data. Each patient was identified using a unique visit ID, and diagnostic information was coded accordingly. If a patient lacked certain laboratory test results, these data were extracted independently by two researchers from clinical records and cross-checked to minimize bias. Finally, the original datasets were merged into a master table, where each row represented a unique patient (patient ID) and each column contained continuous and categorical variables describing patient characteristics.

#### 2.2.2 Treatment of outcome indicators

In this study, indicators related to blood glucose fluctuations during hospitalization were defined as research endpoints. Based on the actual measurement time points of patients, and combined with the hospital’s work and rest schedule, we summarized the time range of multiple daily measurements of patients as follows: post-breakfast (05:00-06:59), the second pre-breakfast segment (07:00-08:59), post-breakfast (09:00-10:59), before lunch (11:00-12:59), post-lunch (13:00-14: 59), post-lunch segment 2 (15:00-16:59), pre-dinner (17:00-18:59), post-dinner (19:00-20:59), pre-bedtime segment 1 (21:00-22:59), pre-bedtime segment 2 (23:00-00:59), early morning segment 1 (01:00-02:59), early morning segment 2 (03:00-04:59).

In the real-world, patients may not be able to measure their fingertip blood glucose every day during their stay in the hospital according to a strict measurement schedule, due to a combination of subjective factors such as patient compliance and a variety of objective factors such as consultative examinations. To minimize data bias, strict inclusion criteria for GV index were formulated (refer to #4 and #5 in the inclusion criteria). TIR (blood glucose range: 3.9-10.0mmol/L) and the time in tight range (TITR) (blood glucose range: 3.9-7.8mmol/L) were calculated based on fingertip glucose values at least 6 times per day as an indicator for assessing daily glucose fluctuations.

Patients with poorly controlled T2DM generally require 7–10 days of SIIT during hospitalization; therefore, achieving TIR ≥ 70% within 10 days was considered the primary outcome. Because TIR only represents the proportion of time with glucose levels between 3.9- 10.0 mmol/L, we also analyzed TITR as a subgroup variable to further assess glycemic control during hospitalization.

#### 2.2.3 Missing value handling

**Figure 1:**
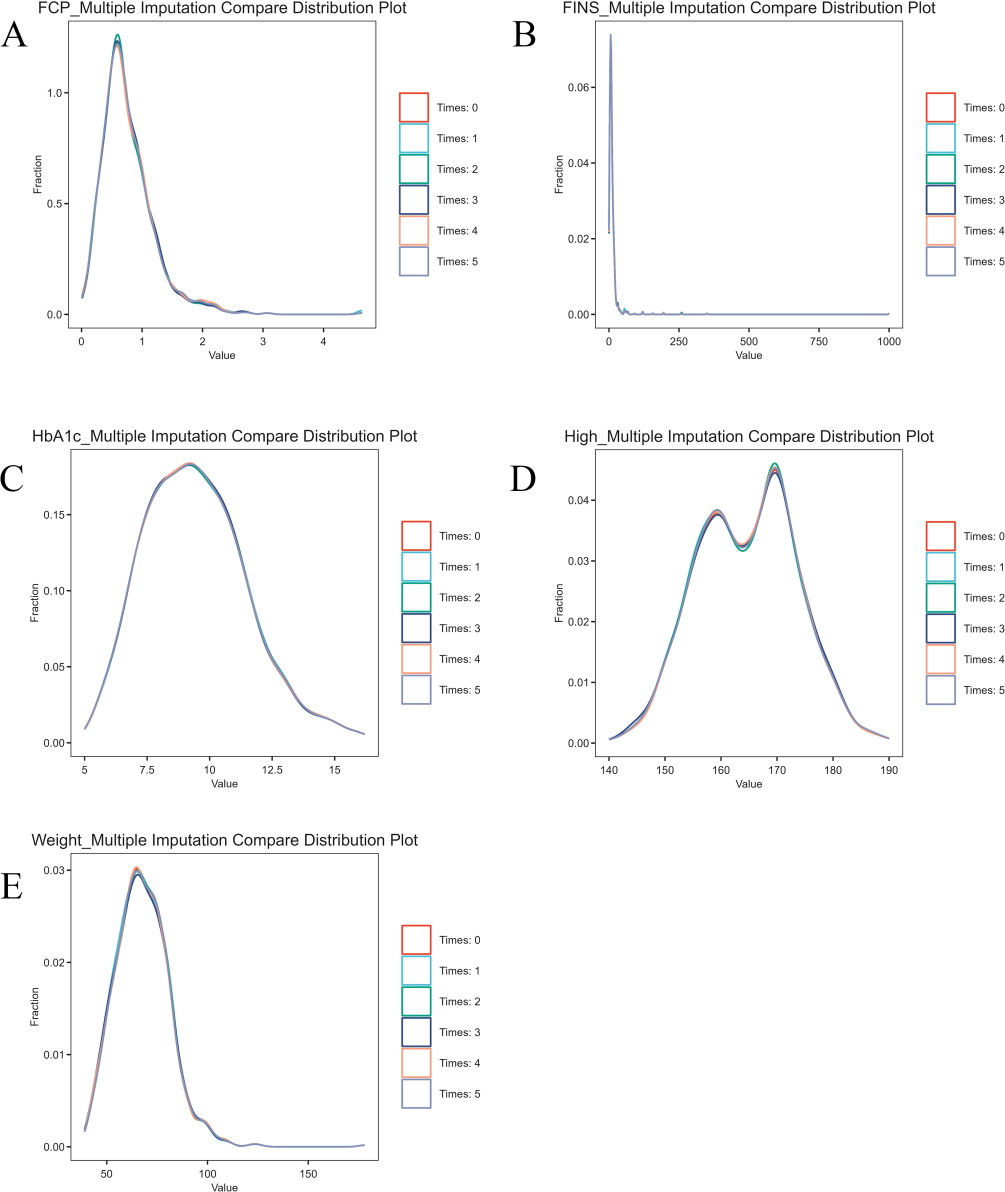
Comparison of multiple imputation results. Comparison of multiple imputation results. Time0 represents the data before imputing missing values, and Time1–Time5 represent the results of the five imputations. **(A)** Comparison of multiple imputations for fasting C-peptide; **(B)** Comparison of multiple imputations for fasting insulin; **(C)** Comparison of multiple imputations for HbA1c; **(D)** Comparison of multiple imputations for height; **(E)** Comparison of multiple imputations for weight.

After manual inquiry and supplementation, height, weight, HbA1c, fasting C-peptide (FCP) and fasting insulin (FINS) were still missing. Because the number of missing values was small, we chose to impute these missing data. To minimize bias, multiple imputation using predictive mean matching was applied for height (36 missing values), weight (32), HbA1c (13), FCP (83), and FINS (67). Continuous variables, including age, sex, length of stay, and FBG, as well as dichotomous variables such as the presence of chronic diabetic complications, obesity, fatty liver, metabolic syndrome, and hypertension, were included in the regression-based imputation model. This procedure generated five separate datasets (five imputations) (**Figure 1**).The consistency of the data before and after imputation was good, indicating that this method ensures the reliability and applicability of the model.

#### 2.2.4 Grouping conditions

Patients were categorized into four groups based on their TIR and time in tighter range (TITR) within the first 10 days of hospitalization. Initially, patients were divided into two main cohorts according to whether they achieved TIR ≥70% during the 10-day treatment period. Subsequently, to further explore the factors associated with stricter glycemic control (3.9–7.8 mmol/L), a subgroup analysis was conducted within the TIR-passing cohort (Cohort 2). Specifically, patients in Cohort 2 were further classified according to whether they achieved both TIR ≥70% and TITR ≥50%. The grouping framework is summarized in **Table 1**.

**Table 1:** Patient grouping overview.

Grouping level	Cohort/Subgroup name	Grouping criteria	Description
Primary grouping (by TIR)	Cohort 1 – TIR failing group	TIR < 70% within 10 days of treatment	Patients who did not achieve TIR ≥70% during the 10-day hospitalization period.
	Cohort 2 – TIR passing group	TIR ≥ 70% within 10 days of treatment	Patients who achieved TIR ≥70% during the 10-day hospitalization period.
Subgroup analysis (within Cohort 2)	Subgroup 1 – TIR ≥70% & TITR ≥50%	TIR ≥70% and TITR ≥50% within 10 days	Patients with both good overall glucose control and stable glucose levels within the tighter range (3.9–7.8 mmol/L).
	Subgroup 2 – TIR ≥70% but TITR <50%	TIR ≥70% but TITR <50% within 10 days	Patients achieving general TIR targets but failing to meet the tighter glucose control standard.

#### 2.2.5 Statistical analysis

All clinically collected variables were analyzed for differences using SPSS version 27, and candidate variables showing significant differences between the two groups (P < 0.05) were selected. Non-normally distributed data were analyzed using the Mann–Whitney U test, and binary categorical variables were compared using the chi-square test. Normally distributed data were expressed as mean ± standard deviation, skewed data as median (interquartile range), and categorical data as frequency (percentage). A P-value of < 0.05 was considered statistically significant.

After identifying the key variables, we constructed both logistic regression and random forest models using the scikit-learn library in Python version 3.11.4. For the logistic regression model, L2 regularization (regularization strength C = 1.0) was applied, and the “lbfgs” optimizer was used for training, with the maximum number of iterations set to 1000. The dataset was randomly split into training and testing sets in a 7:3 ratio, with a random seed of 42 to ensure reproducibility.

For the random forest model, an ensemble learning approach was adopted by constructing multiple decision trees for prediction. The random seed was also set to 42 to maintain consistency and reproducibility. The dataset for this model was randomly divided into training and testing sets in an 8:2 ratio, and the SMOTE technique was applied to address the issue of class imbalance.

The performance of both models was evaluated using multiple metrics, including the area under the receiver operating characteristic (ROC) curve (AUC), calibration curves, the Hosmer–Lemeshow goodness-of-fit test, and decision curve analysis (DCA). After assessing the performance of both models, the logistic regression model was selected as the final model for statistical analysis. To further verify the model’s reproducibility, the logistic regression model was revalidated using Zstats 2025 and R version 4.5.1. In addition, the model’s performance was comprehensively evaluated based on the F1 score, recall, specificity, accuracy, and AUC.

## 3 Results

### 3.1 Baseline characteristics

**Figure 2:**
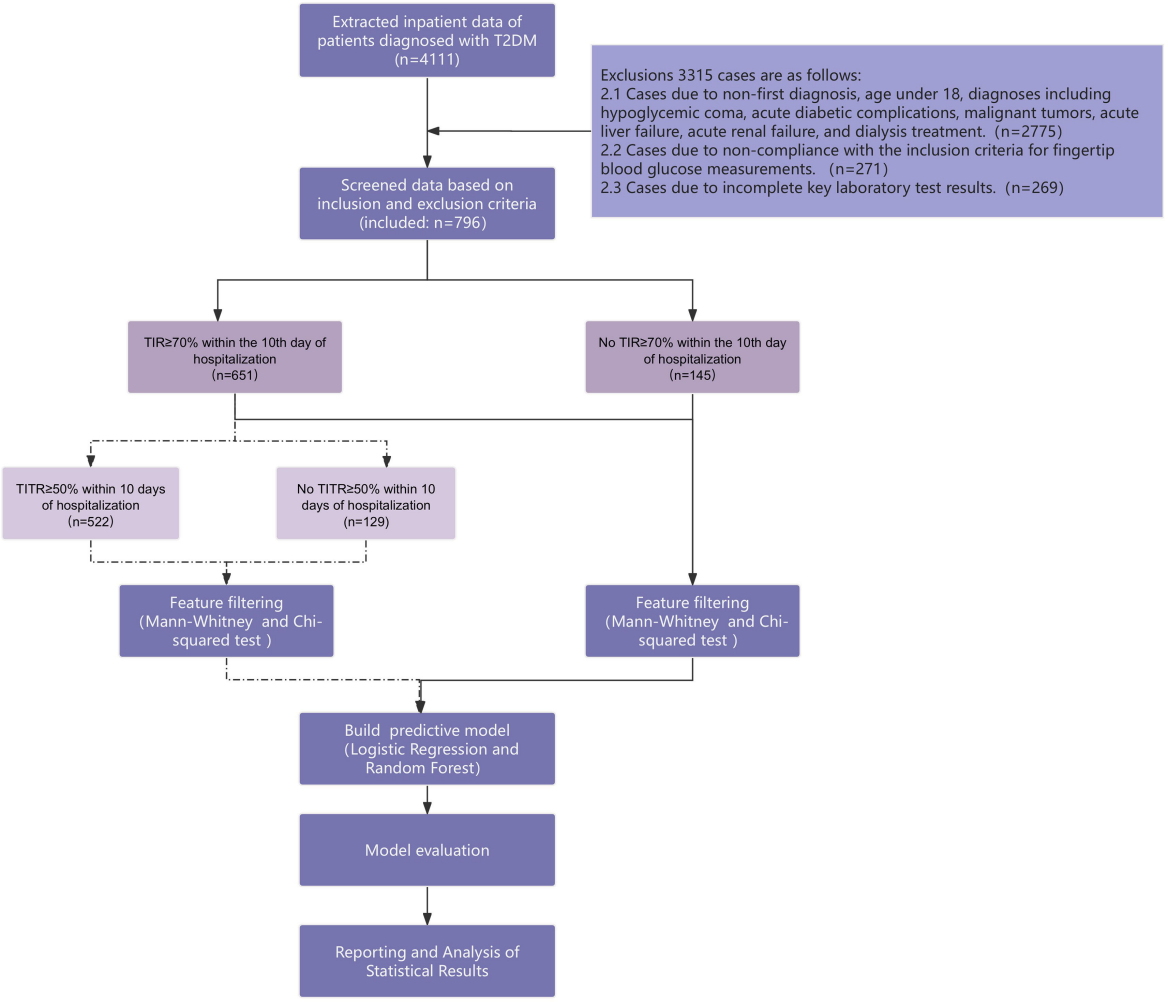
Research flow chart.

As shown in **Figure 2**, a total of 796 patients with type 2 diabetes were included in this study according to the inclusion and exclusion criteria. **Table 2** summarizes their basic characteristics, including age at admission and length of hospital stay., the mean HbA1c within the two months prior to admission was 9.48%. The mean age was 63.01 (SD = 13.48), including 463 males and 333 females. The average length of hospitalization was 13.02 days. Cohorts 1 (no TIR ≥ 70% event occurred within 10 days of hospitalization) included 145 people (97 males and 48 females), aged between 21 and 92 years, with a median age of 67 years. The median length of hospitalization was 14 days (Q1 = 10 days, Q3 = 17 days). A total of 651 people (366 males and 285 females) in Cohorts 2 (TIR ≥ 70% events occurred within 10 days of hospitalization) were aged between 20 and 91 years, with a median age of 64 years. The median length of hospitalization was 12 days (Q1 = 10 days, Q3 = 15 days). The participant inclusion process is shown in **Figure 2** (**Supplementary Table 1**).

**Table 2:** Comparison of baseline clinical characteristics and laboratory parameters between the two cohorts.

Variable	Levels	Overall	Cohorts 1	Cohorts 2
		N = 796	N = 145	N = 651
Length of hospital stay		13.02 ± 4.18	14 (10 – 17)	12 (10– 15)
Age		63.01 ± 13.48	67 (59 – 77)	64 (54 – 72)
Sex				
	Male	463 (58.17%)	97 (66.90%)	366 (56.22%)
	Female	333 (41.83%)	48 (33.10%)	285 (43.78%)
High		1.65 ± 0.09	166 (159 – 172)	165 (158 – 170)
Weight		67.83 ± 13.42	65 (58.00 - 75.00)	67 (59.00 - 75.00)
BMI		24.91 ± 3.87	23.88 (21.64 - 26.64)	25 (22.48 - 27.04)
FBG		9 ± 3.47	9.56 (7.81 - 12.40)	8.22 (6.27 - 10.55)
HbA1c		9.48 ± 2.06	9.7 (8.40 - 11.22)	9.2 (7.80 - 10.70)
FIN		13 ± 40.46	8.5 (4.90 - 13.60)	8.1 (4.90 - 12.60)
FCP		0.77 ± 0.44	0.68 (0.47 - 1.00)	0.68 (0.50 - 0.96)
TG		2.05 ± 3.17	1.39 (0.92 - 1.97)	1.46 (1.05 - 2.19)
TC		1.04 ± 0.44	0.99 (0.79 - 1.25)	0.95 (0.83 - 1.16)
LDL		2.53 ± 0.89	2.31 (1.76 - 2.92)	2.5 (1.93 - 3.13)
HDL		1.04 ± 0.44	0.99 (0.79 - 1.25)	0.95 (0.83 - 1.16)
Neutrophils		4.13 ± 2.02	3.7 (2.80 - 4.90)	3.7 (2.90 - 4.80)
Lymphocyte		1.84 ± 0.66	1.6 (1.30 - 1.90)	1.8 (1.40 - 2.30)
NLR		2.66 ± 2.3	2.17 (1.63 - 3.23)	2 (1.50 - 2.80)
TyG		9.24 ± 0.84	9.33 (8.77 - 9.74)	9.17 (8.67 - 9.72)

BMI, Body mass index; FIN, Fasting insulin;TyG, Triglyceride and Glucose Index;FINS, Fasting Insulin;FCP, Fasting C-peptide;NLR, Neutrophil to Lymphocyte Ratio; HbA1c, Glycated hemoglobin.

At the same time, to observe stricter glycemic control, patients in Cohort 2 were divided into two subgroups according to whether they achieved TITR ≥ 50%. Subgroup 1 included patients without TITR ≥ 50% (n = 129), and Subgroup 2 included those with TITR ≥ 50% (n = 522). A subgroup analysis was then performed. In Subgroup 1, the proportion of males was higher than females (62.02% vs. 37.98%), the median age was 65 years, and the median HbA1c was 9.70%. In Subgroup 2, the proportions of males and females were similar (54.79% vs. 45.21%), with a median age of 63 years and a median HbA1c of 9.10% (**Supplementary Table 2**).

### 3.2 GV index characteristics

**Figure 3:**
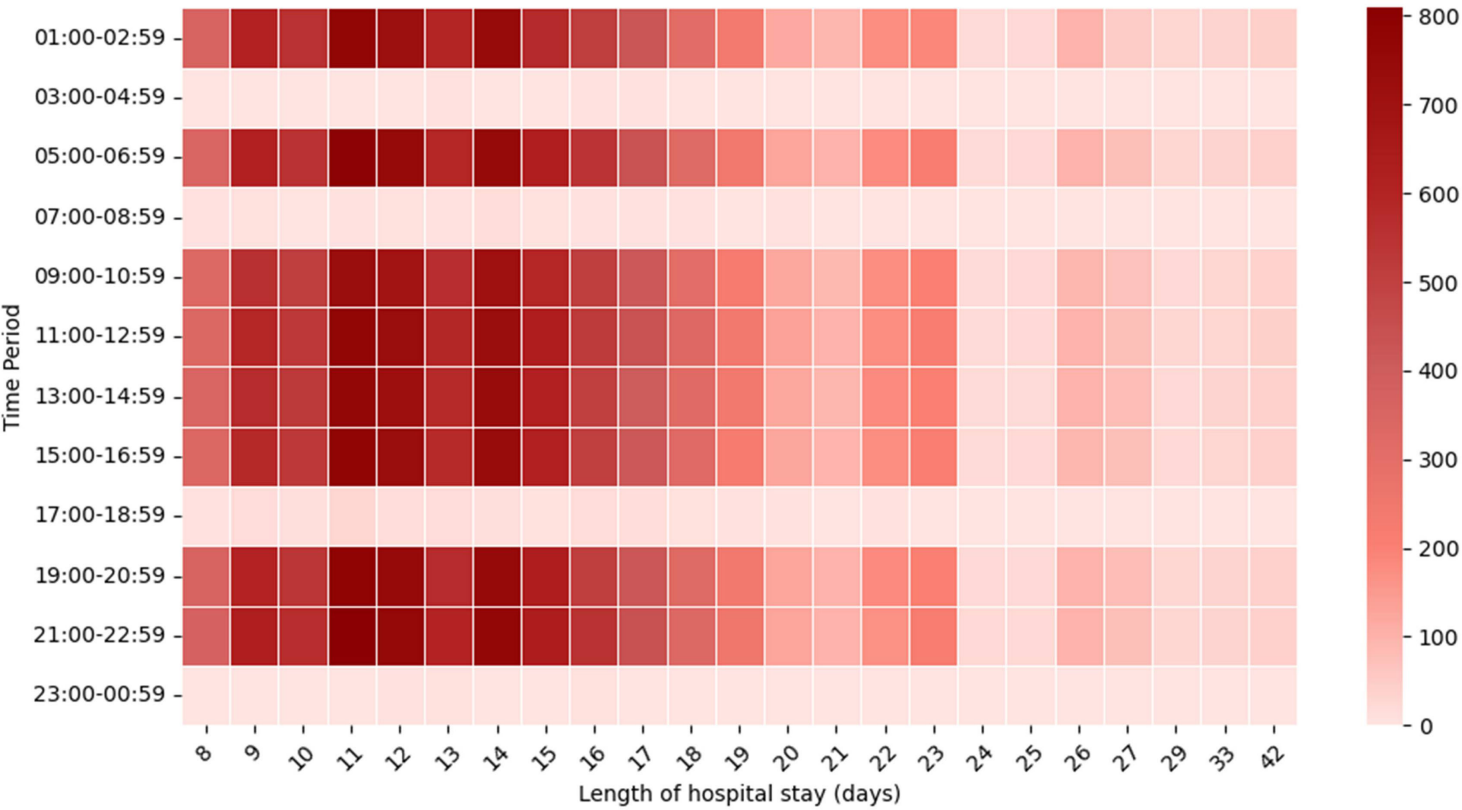
Fingertip blood glucose monitoring time distribution heat map. Distribution of blood glucose measurements during hospitalization. Each two-hour interval is defined as a measurement time period (y-axis), and hospital days are shown on the x-axis. The total sum of blood glucose values within each time period is plotted, with darker colors indicating higher total glucose values.

In the patient population with different hospitalization days, fingertip blood glucose measurements were concentrated across eight time periods covering 24 hours: 01:00-02:59; 05:00-06:59; 09:00-10:59; 11:00-12:59; 13:00-14:59; 15:00-16:59; 19:00-20:59 and 21:00-22:59. This finding indicates that TIR and TITR values calculated from multiple daily fingertip glucose measurements can effectively reflect each patient’s glycemic control within the same day (**Figure 3**) (**Supplementary Figure 1**).

**Figure 4:**
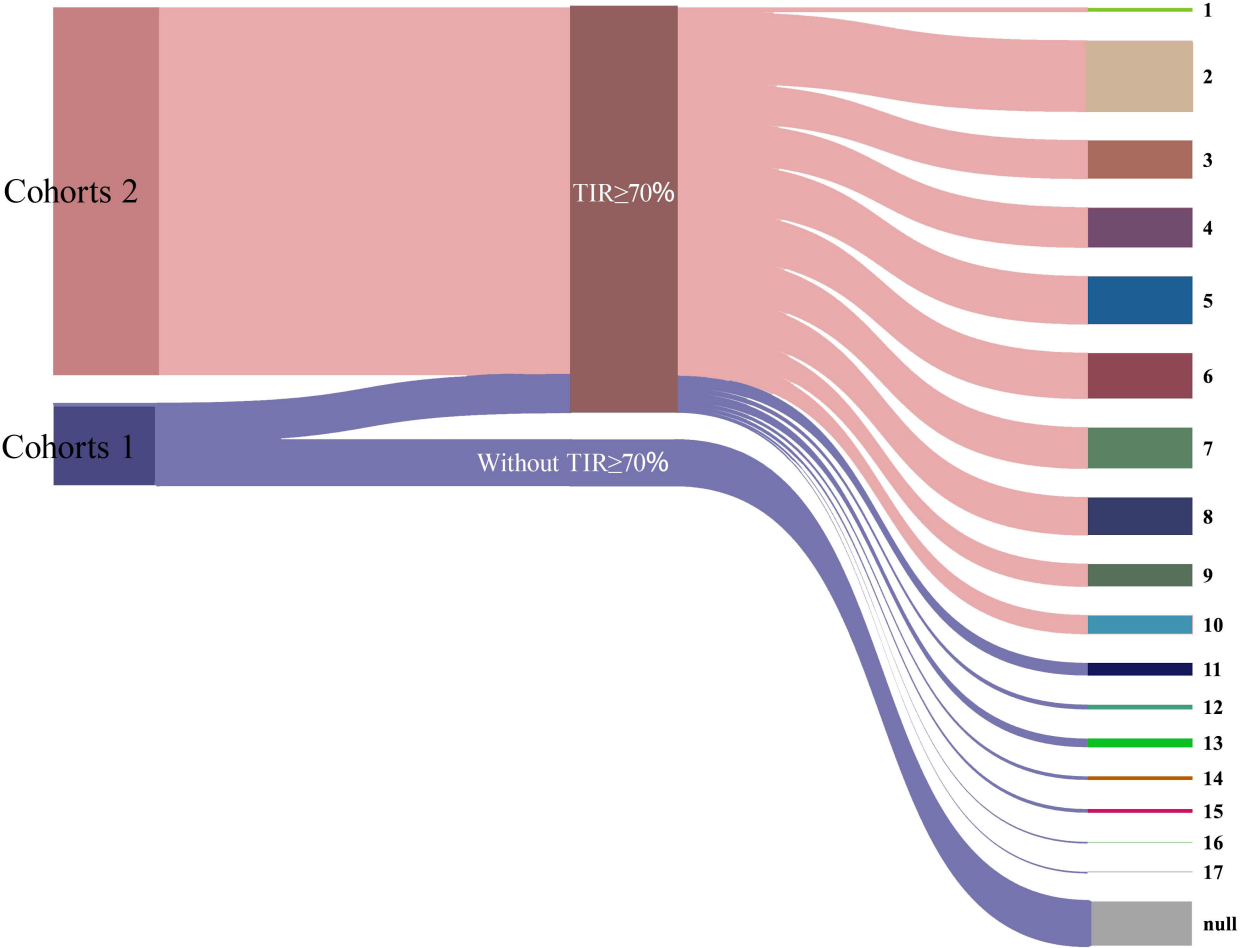
First TIR ≥ 70% of hospitalization days. The thickness of the stripes represents the number of individuals, and the values on the right indicate the total number of hospitalization days. “Null” denotes that TIR ≥ 70% was not achieved during the hospitalization period.

The length of hospitalization and number of people who achieved TIR ≥70% for the first time in Cohorts 1 and Cohorts 2 were counted. In Cohort 2 (patients who achieved TIR ≥ 70% within 10 days of hospitalization), most people achieved TIR ≥ 70% on the second day of hospitalization (n=126). After that, the number of people who achieved TIR ≥ 70% within 3–8 days of hospitalization fluctuated between 67-85, which was relatively stable. In Cohort 1 (patients who did not achieve TIR ≥ 70% within 10 days), most reached the target on day 11 (n = 23) or day 13 (n = 16). Additionally, 80 patients did not achieve TIR ≥ 70% at any point during hospitalization (**Figure 4**).

### 3.3 Screening of differential variables and model construction

#### 3.3.1 Differential variables between the 2 cohorts

**Figure 5:**
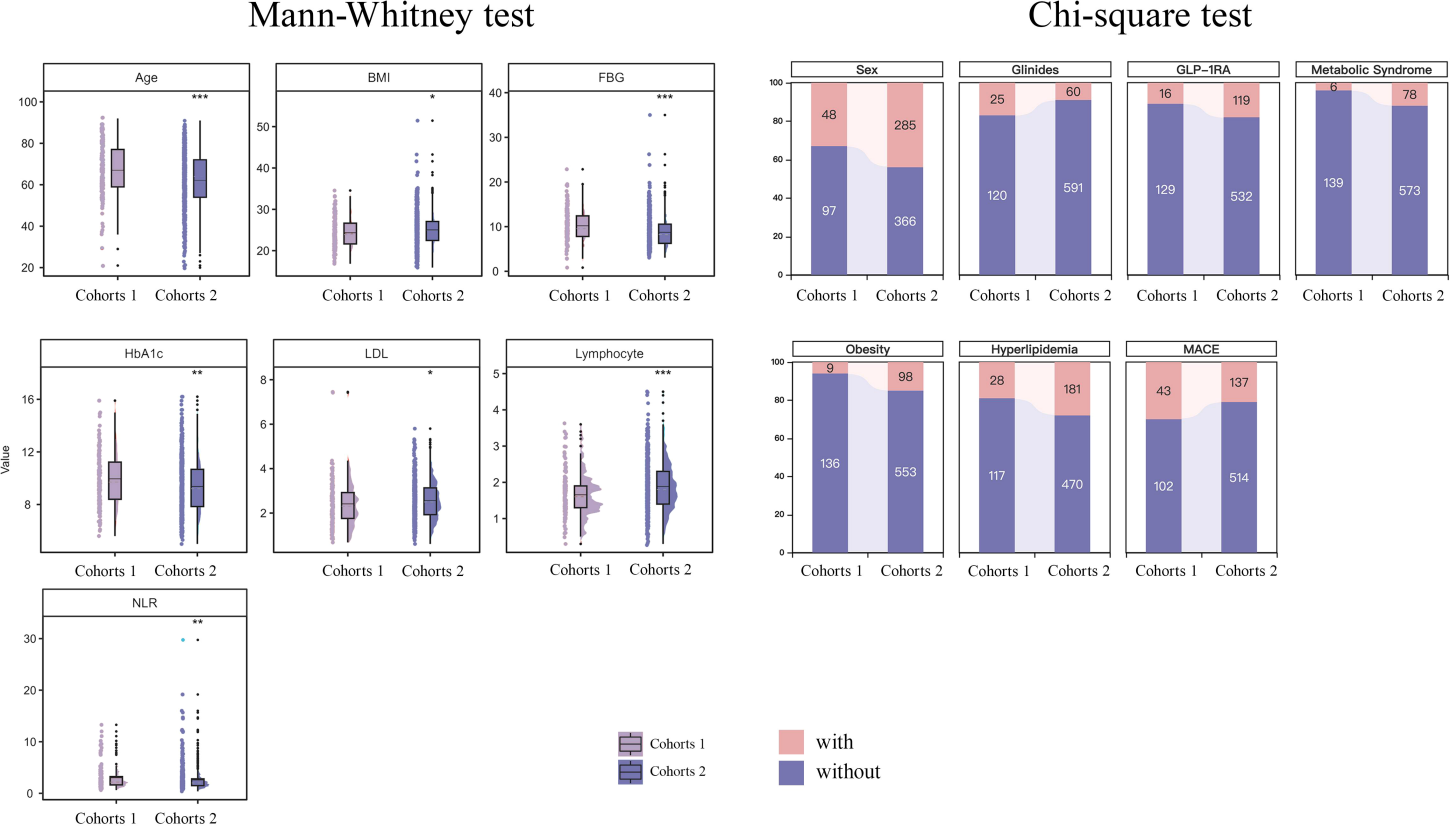
Difference variable between the two cohorts. Cohort 1 included patients who did not experience a TIR ≥ 70% event within the first 10 days of hospitalization. Cohort 2 included those who did experience a TIR ≥ 70% event within the same period.

Statistical analysis of the differential characteristics between Cohort 1 and Cohort 2 revealed significant differences (P < 0.05) in the following variables: age, sex, FBG, HbA1c, low-density lipoprotein (LDL), lymphocyte count, neutrophil-to-lymphocyte ratio (NLR), body mass index (BMI), glucagon-like peptide-1 receptor agonist (GLP-1RA) use, glinide use, hyperlipidemia, obesity, metabolic syndrome, and major adverse cardiac events (MACE) (**Figure 5**).

#### 3.3.2 Differential variables between the 2 subgroups

**Figure 6:**
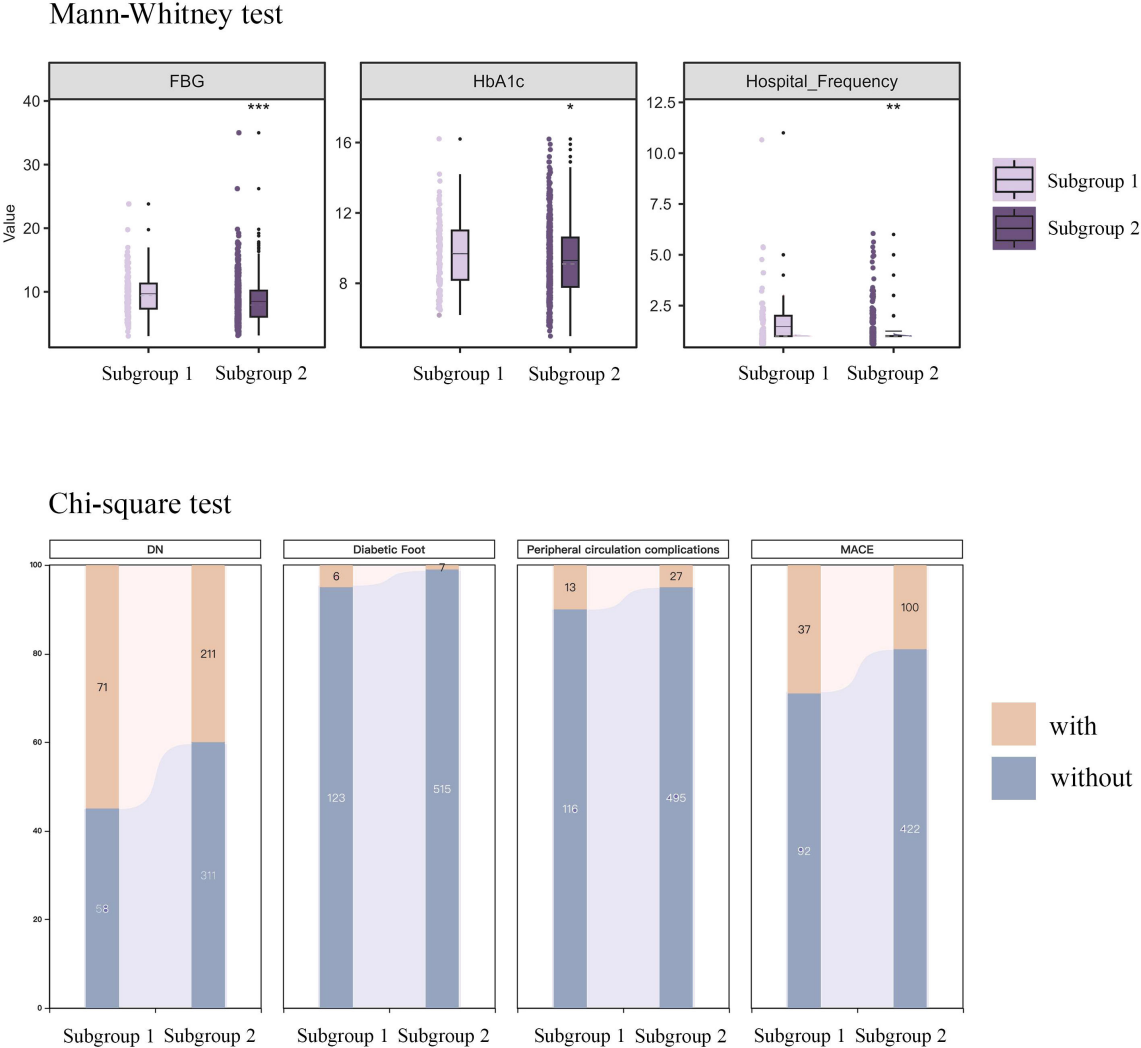
Difference variable between the two subgroup. Subgroup 1: No TITR ≥50% event occurred. Subgroup 2: TITR ≥50% event occurred.

Statistical analysis of the differential characteristics between the 2 subgroups revealed significant differences (P < 0.05) in the following variables: FBG, HbA1c, DN, diabetic foot, peripheral circulation complications, and MACE (**Figure 6**).

#### 3.3.3 Model construction and evaluation

Using the differential characteristics between the 2 cohorts, age, BMI, FBG, HbA1c, LDL, lymphocyte, NLR, sex, glinides, GLP-1RA, metabolic syndrome, obesity, hyperlipidemia, and MACE as feature variables, we constructed a logistic regression model (Model LR1) and a random forest model (Model RT1) to predict whether a TIR ≥ 70% event would occur within the 10-day hospitalization period.

Both models showed good predictive performance (Model LR1 AUC = 0.81; F1 score = 0.89; Model RT1 AUC = 0.80, F1 score = 0.89). However, due to class imbalance, both models exhibited poor predictive performance for patients who did not achieve TIR ≥ 70% events within the 10-day hospitalization period (Model LR1 Specificity = 0.07; Model RT1 Specificity = 0.02). After applying undersampling to correct this imbalance, specificity improved markedly in both models (to 0.54).Moreover, after undersampling adjustment, Model LR1 had higher F1 score and accuracy compared to Model RT1, indicating that logistic regression provided better predictive stability (**Table 3**).

**Table 3:** Evaluation results of different models.

Algorithm	Logistic regression		Random forest		Logistic regression		Random forest	
Sampling	Full	Under	Full	Under	Full	Under	Full	Under
	Model LR1		Model RT1		Model LR2		Model RT2	
Accuracy	0.81	0.63	0.80	0.59	0.80	0.59	0.72	0.58
Recall	0.98	0.85	0.98	0.75	0.99	0.66	0.88	0.50
Precision	0.82	0.57	0.81	0.54	0.80	0.57	0.79	0.58
Specificity	0.07	0.45	0.02	0.45	0.03	0.53	0.10	0.65
F1 Score	0.89	0.68	0.89	0.63	0.89	0.61	0.84	0.54

Using the differential characteristics between the 2 subgroups, namely HbA1c, FBG, DN, diabetic foot, peripheral circulation complications, and MACE as feature variables, we constructed a logistic regression model (Model LR2) and a random forest model (Model RT2) to predict whether a TITR ≥ 50% event would occur within the 10-day hospitalization period for patients achieving a TIR ≥ 70%.

Both models performed well overall(Model LR2 AUC = 0.80; F1 score = 0.89; Model RT2 AUC = 0.72, F1 score = 0.84). However, class imbalance again resulted in poor specificity for patients who did not achieve both TIR ≥ 70% and TITR ≥ 50% (Model LR2 specificity = 0.03; Model RT2 specificity = 0.10). After undersampling, specificity increased significantly (Model LR2 = 0.53; Model RT2 = 0.65). Furthermore, Model LR2 maintained higher F1 score and accuracy than Model RT2, suggesting that logistic regression remained more robust for model construction (**Table 3**).

**Figure 7:**
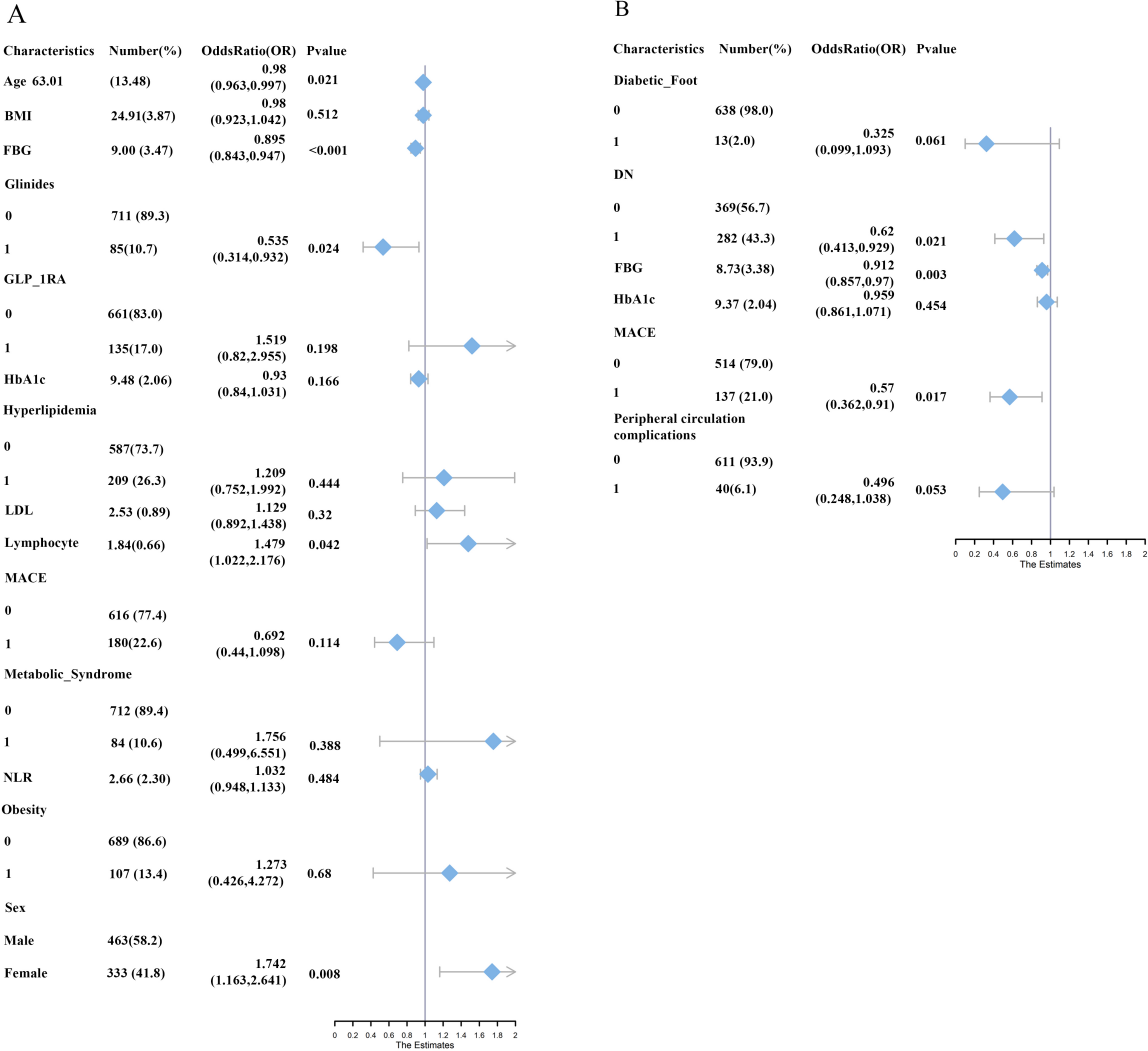
**(A)** The variable forest plot for Model LR1; **(B)** The variable forest plot for Model LR2. In the figures, “0” indicates the absence of the diagnosis or non-use of the oral hypoglycemic agent, while “1” indicates the presence of the diagnosis or use of the oral hypoglycemic agent.

**Figure 8:**
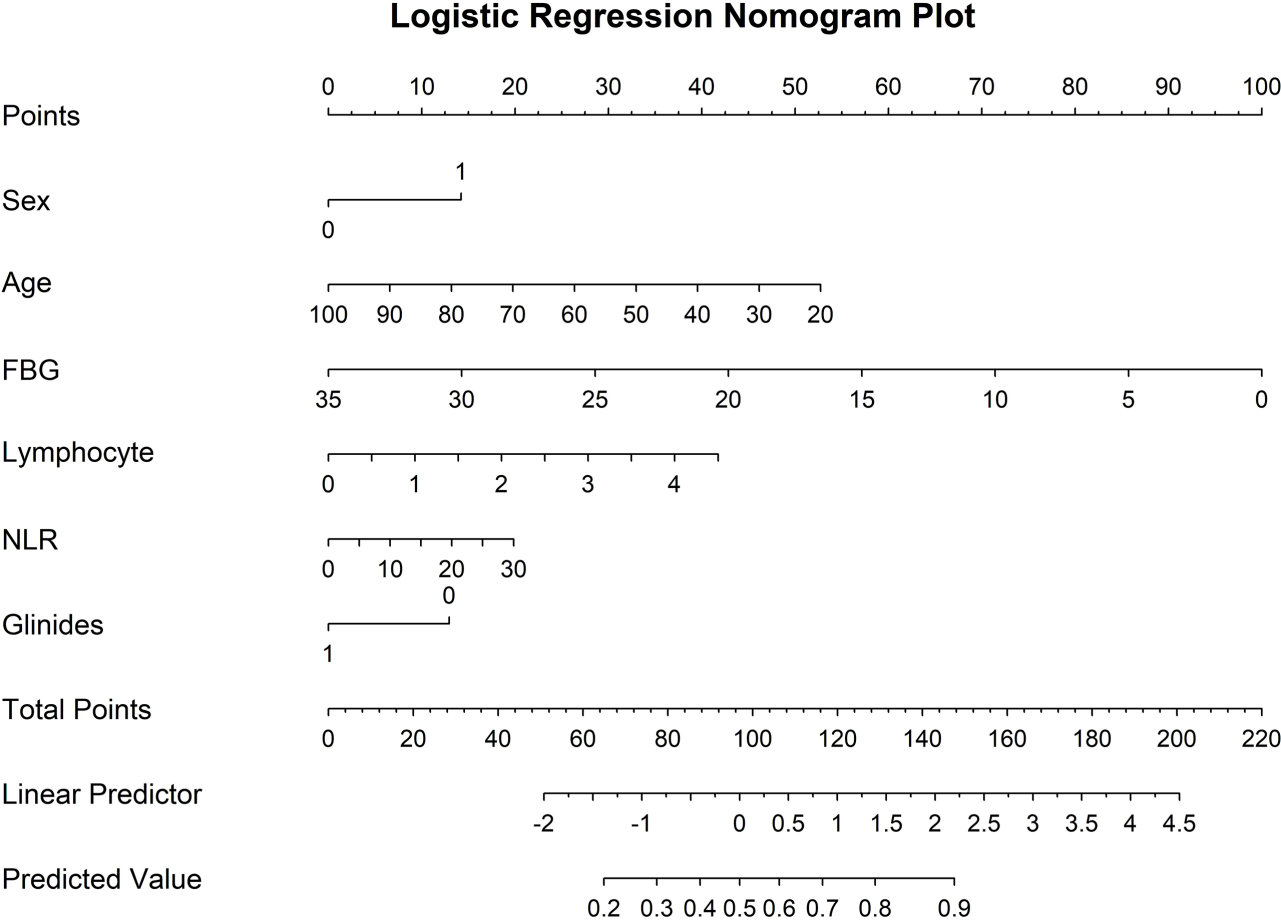
Logistic Regression Nomogram Plot. Based on the final predictive model, a nomogram was constructed to estimate the probability of achieving the target TIR during hospitalization. Variables were coded as follows: Sex (0: male; 1: female) and Glinides (0: without Glinides; 1: with Glinides).

In Model LR1, female sex, age, FBG, use of glinides, and lymphocyte count were significant contributors (all P < 0.05) (**Figure 7**). Higher age (OR = 0.98, 95% CI: 0.963-0.997), FBG (OR = 0.895, 95% CI: 0.844-0.948), and the use of glinides (OR = 0.535, 95% CI: 0.312-0.92) were negatively associated with achieving TIR ≥ 70% within the 10-day hospitalization period, while female (OR = 1.742, 95% CI: 1.157-2.624) and higher lymphocyte counts (OR = 1.479, 95% CI: 1.014-2.157) were positively associated. A nomogram was constructed based on these factors (**Figure 8**).

In Model LR2, FBG, diabetic nephropathy (DN), and MACE were significant predictors (all P < 0.05) (**Figure 7**). Increases in FBG (OR = 0.912, 95% CI: 0.858-0.97), the presence of DN (OR = 0.62, 95% CI: 0.414-0.929), and occurrence of MACE (OR = 0.57, 95% CI: 0.36-0.903) were negatively associated with achieving TIR ≥ 70% and TITR ≥ 50% within the 10-day hospitalization period.

## 4 Discussion

From a clinical perspective, most patients undergoing SIIT during hospitalization achieved effective glycemic control, with 81.8% achieving TIR ≥ 70% within the 10-day hospitalization period, and 65.6% experiencing stringent control (TIR ≥ 70% and TITR ≥ 50%). However, it is noteworthy that around 10% of patients did not meet the TIR ≥ 70% standard during hospitalization.

This study identified age, sex, FBG, HbA1c, LDL, lymphocyte count, NLR, BMI, GLP-1RA, glinides, hyperlipidemia, obesity, metabolic syndrome, and MACE as significant variables influencing TIR outcomes (TIR ≥ 70%) within the 10-day hospitalization period. During model construction, older age, higher FBG, and the use of glinides were negatively associated with achieving a TIR ≥ 70% during hospitalization. In contrast, female sex and higher lymphocyte counts were positively associated with reaching this target.

HbA1c is currently considered a key biomarker for diabetic complications. However, in this study, it was not significant in the model, suggesting that it may not be a major factor influencing glycemic control during SIIT hospitalization. These findings suggest that HbA1c alone is insufficient for evaluating in-hospital glycemic control.

Notably, an increase in lymphocyte count was positively associated with achieving TIR targets during hospitalization. T2DM is a metabolic disease characterized by an important inflammatory background, and lymphocytes play a complex regulatory role in the pathogenesis of T2DM (15). Based on surface markers and functions, lymphocytes can be divided into three major categories: T cells, B cells, and natural killer (NK) cells, each containing multiple subpopulations that participate differently in inflammatory responses. For example, the overexpression of Th17 cells in T cells is associated with T2DM. The cytokines they secrete, such as IL-17A and IL-22, promote insulin resistance and adipose tissue inflammation (16). Studies have shown that the circulating levels of IL-17 are significantly elevated in obese women and diabetic patients. In contrast, regulatory T cells (Tregs) tend to decrease in obese or T2DM patients. These cells can suppress immune responses through the secretion of IL-10 and TGF-β, or via cell-to-cell contact mechanisms, thereby alleviating inflammatory damage (17). A meta-analysis showed that in patients with type 2 diabetes mellitus (T2DM), the proportion of Th17 cells is elevated whereas that of Treg cells is reduced (18). Meanwhile, several studies have reported that the NK cell counts in T2DM patients are lower than in healthy controls (19, 20), and NK cell activity shows a significant negative correlation with fasting blood glucose, HbA1c, and 2-hour post-load glucose levels. This dysfunction may be related to high Tim-3 expression on NK cells, which leads to impaired NK cell activity and apoptosis (21).

In summary, lymphocyte subpopulation imbalance in T2DM is characterized by overactivation of pro-inflammatory subsets (Th17, B2) and functional suppression of anti-inflammatory subsets (Treg, Breg). These changes collectively drive chronic inflammation, insulin resistance, and β-cell dysfunction. Although different subpopulations of lymphocytes have different mechanisms in the development of T2DM, Allahyani et al. reported that the total lymphocyte proportion in T2DM patients was markedly lower than in healthy individuals (22). Lymphocytes are closely related to the infection risk, poor prognosis (23), and disease severity of patients with T2DM. Further investigation into how specific lymphocyte subsets influence TIR may help identify new therapeutic targets for stabilizing glycemic variability.

In addition, NLR as a novel composite indicator reflecting the inflammatory status of patients with type 2 diabetes mellitus (24), has been identified in previous studies as one of the important factors influencing the achievement of TIR targets. However, in this study, lymphocyte count had a higher weighting and was already included as an independent variable. Since NLR represents the ratio of neutrophil to lymphocyte counts, these two variables are highly collinear. Therefore, to avoid multicollinearity issues, NLR was not included in the model. Moreover, the relatively limited sample size in this study also restricted further analysis of NLR. Nevertheless, NLR remains an important inflammatory marker that warrants clinical attention. High NLR is strongly associated with insulin resistance (25) and have been shown to have significant predictive value for the severity of coronary artery lesions in T2DM patients (26), as well as the diagnosis of microvascular complications and all-cause mortality (27, 28). This could be attributed to the fact that a high NLR often indicates the presence of an inflammatory response, which promotes insulin resistance and necessitates higher insulin doses to maintain glycemic control. Relevant meta-analysis have shown that anti-inflammatory therapies, including anti-IL-1 therapy, can significantly reduce the levels of FPG, HbA1c in patients with T2DM (29). In addition to the commonly used hypoglycemic drugs, butterfly pea flower (Clitoria ternatea L.) (30), berberine (BBR) (31) etc., have also been proven to alleviate chronic inflammation and improve glycemic control. In conclusion, controlling inflammation may help improve the attainment of TIR standards. Monitoring NLR and the number of lymphocytes can help adjust the treatment plan in a timely manner and increase the rate of achieving TIR standards.

In terms of medication use, the findings of this study align with previous research indicating that the use of GLP-1RA and glinides influences inpatient blood glucose management. Several studies have highlighted the efficacy of GLP-1RA in reducing postprandial glucose peaks and overall glycemic variability. This study found that the use of GLP-1RA was a factor affecting target achievement for TIR within the 10-day hospitalization period, although it did not achieve significance in model construction. This may be due to the fact that the improvements in FBG and overall glycemic variability induced by GLP-1RA may require a longer timeframe, and the limitations imposed by the 10-day hospitalization window might not allow the drug to achieve its full effect.

Additionally, the study revealed a negative correlation between the use of glinides and TIR achievement within 10 days. Previous studies have suggested that the use of glinide drugs may increase the risk of hypoglycemia. In this study, 796 patients were analyzed based on whether they used glinides. There were no significant differences between the two groups in TBR, HbA1c, or FBG (P > 0.05). However, significant differences were observed in age, length of hospital stay, types of insulin used (use of ultra-short-acting, short-acting, intermediate-acting, premixed, or long-acting insulin), number of oral antidiabetic drugs, and presence of peripheral vascular complications (**Supplementary Table 4**).To further explore the relationship between glinide use and TIR, a multivariable logistic regression analysis was conducted. The dependent variable (Y) was failure to achieve TIR ≥ 70% (0 = achieved, 1 = not achieved). The main independent variable (X) was the use of glinides. Confounding variables included age, length of hospital stay, types of insulin used, number of oral antidiabetic drugs, and presence of peripheral vascular complications. After adjusting for age, insulin regimen, and other potential confounders, glinide use was not identified as an independent factor associated with failure to achieve TIR ≥ 70% within 10 days of hospitalization (P > 0.05). The difference in TIR achievement observed in the univariate analysis between the glinide and non-glinide groups was mainly due to the imbalance in age distribution and insulin treatment regimens. Nevertheless, for patients receiving intensive insulin therapy, careful blood glucose monitoring remains essential when glinides are prescribed.

Age and sex emerged as key indicators influencing blood glucose variability during hospitalization. Age is not only a significant factor affecting hospitalization rates in T2DM patients but also a critical reason for declines in insulin secretion and sensitivity (32, 33). Furthermore, multiple studies indicate that older T2DM patients exhibit an increased risk of hypoglycemia, resulting in a call amongst experts to relax glycemic control targets for these patients (34, 35). This may lead clinicians to adjust insulin dosages for older patients more cautiously.

In this model, being male also posed a risk factor for not achieving target blood glucose control. To date, various studies have produced inconsistent results regarding sex differences in blood glucose control. A 2013 meta-analysis indicated that males tended to achieve HbA1c levels of ≤ 7% more easily (2). However, female patients exhibited more significant reductions in FPG (36). A retrospective study in the UK involving 6,032 T2DM patients receiving insulin therapy found that age and BMI were key determinants of insulin treatment outcomes, while sex was not a critical predictive factor (37). The inconsistent results regarding sex in T2DM may be attributed to different environmental factors or insulin regimens, requiring further validation through larger retrospective studies.

Furthermore, TBR, as an indicator of hypoglycemia, is often considered to be associated with TIR. As shown in **Supplementary Table 3**, patients were divided into two groups according to whether TBR events occurred during hospitalization (125 patients with TBR events and 671 without). We found that TBR was not significantly correlated with the attainment of TIR, but was closely associated with body weight, age, fasting C-peptide levels, and hypertension.

This finding suggests that hypoglycemia during SIIT may be more strongly influenced by individual metabolic status and insulin sensitivity rather than overall glycemic stability. Older age, lower body weight, and impaired *β*-cell function (reflected by reduced fasting C-peptide) may predispose patients to hypoglycemia even when their TIR remains within target levels. Therefore, clinicians should balance intensive glycemic control with the risk of hypoglycemia and consider moderately relaxing glycemic targets in such patients to help prevent TBR events.

In the subsequent subgroup analyses, FBG, HbA1c, DN, diabetic foot, peripheral circulation complications, and MACE were identified as indicators influencing the outcomes. This suggests that the occurrence of complications has a significant impact under stricter glycemic control. In the model construction, a history of FBG, DN, and MACE was incorporated as relevant factors for the model.

DN, as the most severe microvascular complication of diabetes, is also a primary cause of end-stage renal disease (ESRD). Previous studies have indicated that patients with DN exhibit significantly lower TIR during insulin therapy compared to those without DN, and that lower TIR is associated with an increased risk of developing DN (38). On the one hand, chronic poor glycemic control is a significant risk factor for the development of DN. On the other hand, renal impairment in patients with DN leads to decreased insulin clearance, predisposing them to hypoglycemia. This often compels clinicians to relax glycemic targets (39). Moreover, the chronic inflammatory state associated with DN, characterized by elevated levels of TNF-α and IL-6, further impairs insulin sensitivity and increases the difficulty of glycemic control. Additionally, no studies have yet explored the TIR in insulin-treated patients with a history of MACE. However, the incidence of MACE is closely related to the level of glycemic control (40), with patients experiencing greater glycemic variability showing a significantly higher incidence of MACE (41).

In summary, DN and MACE exacerbate the difficulty of glycemic control through multiple pathophysiological mechanisms, including insulin resistance, inflammation, and oxidative stress. Conversely, chronic hyperglycemia accelerates the progression of complications. In the context of strict glycemic control, the presence of pre-existing complications demonstrates a stronger correlation.

Research on factors influencing TIR and other glycemic variability indices in T2DM patients undergoing SIIT remains limited. The findings of this study may help clinicians better understand blood glucose regulation in SIIT patients and guide adjustments to treatment strategies. However, this study has several limitations. Firstly, it is a single-center retrospective analysis, and the data do not encompass diverse subpopulations with varying disease durations, age groups, and complication severities, potentially leading to a bias in results. While some confounding variables were adjusted, unmeasured confounders (such as dietary patterns and medication adherence) could influence the results. Secondly, there is still room for optimization in the model to meet clinical precision decision-making needs. Future studies should broaden data sources and construct composite endpoints by integrating TIR, TBR, and glycemic fluctuations to provide more robust support for predicting blood glucose fluctuation during hospitalization.

## 5 Conclusion

As a standard treatment regimen for T2DM, SIIT effectively lowers blood glucose levels and reduces the risk of acute complications. Although TIR has emerged as a key indicator in diabetes management, it remains underutilized in clinical practice.

In this study, we developed a machine learning–based predictive model to estimate the likelihood of achieving TIR targets during hospitalization among T2DM patients receiving SIIT. This model can help clinicians identify patients at risk of poor glycemic control, enabling timely adjustment of insulin regimens and personalized management to improve clinical outcomes.

Moreover, it provides a data-driven foundation for identifying key determinants of glycemic variability. From a mechanistic perspective, investigating TIR-related factors may help reveal the underlying relationships between glycemic stability, inflammatory responses, β-cell recovery, and metabolic regulation in T2DM, thereby offering new insights for precision interventions.

## Data Availability

The original contributions presented in the study are included in the article/**Supplementary Material**. Further inquiries can be directed to the corresponding authors.

## References

[1] Wang L Peng W Zhao Z Zhang M Shi Z Song Z . Prevalence and treatment of diabetes in China, 2013-2018. Jama. (2021) 326:2498–506. doi: 10.1001/jama.2021.22208, PMID: 34962526 PMC8715349

[2] McGill JB Vlajnic A Knutsen PG Recklein C Rimler M Fisher SJ . Effect of gender on treatment outcomes in type 2 diabetes mellitus. Diabetes Res Clin Pract. (2013) 102:167–74. doi: 10.1016/j.diabres.2013.10.001, PMID: 24183259

[3] Adler AI Coleman RL Leal J Whiteley WN Clarke P Holman RR . Post-trial monitoring of a randomised controlled trial of intensive glycaemic control in type 2 diabetes extended from 10 years to 24 years (UKPDS 91). Lancet. (2024) 404:145–55. doi: 10.1016/S0140-6736(24)00537-3, PMID: 38772405

[4] Mingrone G Panunzi S De Gaetano A Guidone C Iaconelli A Capristo E . Metabolic surgery versus conventional medical therapy in patients with type 2 diabetes: 10-year follow-up of an open-label, single-centre, randomised controlled trial. Lancet. (2021) 397:293–304. doi: 10.1016/S0140-6736(20)32649-0, PMID: 33485454

[5] Weng J Li Y Xu W Shi L Zhang Q Zhu D . Effect of intensive insulin therapy on beta-cell function and glycaemic control in patients with newly diagnosed type 2 diabetes: a multicentre randomised parallel-group trial. Lancet. (2008) 371:1753–60. doi: 10.1016/S0140-6736(08)60762-X, PMID: 18502299

[6] Li Y Xu W Liao Z Yao B Chen X Huang Z . Induction of long-term glycemic control in newly diagnosed type 2 diabetic patients is associated with improvement of beta-cell function. Diabetes Care. (2004) 27:2597–602. doi: 10.2337/diacare.27.11.2597, PMID: 15504992

[7] Kramer CK Zinman B Choi H Retnakaran R . Predictors of sustained drug-free diabetes remission over 48 weeks following short-term intensive insulin therapy in early type 2 diabetes. BMJ Open Diabetes Res Care. (2016) 4:e000270. doi: 10.1136/bmjdrc-2016-000270, PMID: 27547422 PMC4985916

[8] Cheng L Yang F Cao X Li GQ Lu TT Zhu YQ . The effect of short-term intensive insulin therapy on circulating T cell subpopulations in patients with newly diagnosed type 2 diabetes mellitus. Diabetes Res Clin Pract. (2019) 149:107–14. doi: 10.1016/j.diabres.2019.02.007, PMID: 30759366

[9] Gomez-Peralta F Choudhary P Cosson E Irace C Rami-Merhar B Seibold A . Understanding the clinical implications of differences between glucose management indicator and glycated haemoglobin. Diabetes Obes Metab. (2022) 24:599–608. doi: 10.1111/dom.14638, PMID: 34984825

[10] Battelino T Danne T Bergenstal RM Amiel SA Beck R Biester T . Clinical targets for continuous glucose monitoring data interpretation: recommendations from the international consensus on time in range. Diabetes Care. (2019) 42:1593–603. doi: 10.2337/dci19-0028, PMID: 31177185 PMC6973648

[11] Lanspa MJ Krinsley JS Hersh AM Wilson EL Holmen JR Orme JF . Percentage of time in range 70 to 139 mg/dL is associated with reduced mortality among critically ill patients receiving IV insulin infusion. Chest. (2019) 156:878–86. doi: 10.1016/j.chest.2019.05.016, PMID: 31201784

[12] Liu L Ke W Xu L Li H Liu J Wan X . Evaluating the role of time in range as a glycemic target during short-term intensive insulin therapy in patients with newly diagnosed type 2 diabetes. J Diabetes. (2023) 15:133–44. doi: 10.1111/1753-0407.13355, PMID: 36650669 PMC9934958

[13] Shang H Qin X Li W Xu Y Wang W . Relationship between the triglyceride glucose index and time in range: A cross-sectional study based on patients with type 2 diabetes mellitus. J Int Med Res. (2025) 53:3000605251381190. doi: 10.1177/03000605251381190, PMID: 41006067 PMC12475644

[14] Sun R Duan Y Zhang Y Feng L Ding B Yan R . Time in range estimation in patients with type 2 diabetes is improved by incorporating fasting and postprandial glucose levels. Diabetes Ther. (2023) 14:1373–86. doi: 10.1007/s13300-023-01432-2, PMID: 37328714 PMC10299970

[15] Ip BC Hogan AE Nikolajczyk BS . Lymphocyte roles in metabolic dysfunction: of men and mice. Trends Endocrinol Metab. (2015) 26:91–100. doi: 10.1016/j.tem.2014.12.001, PMID: 25573740 PMC4315738

[16] Abdel-Moneim A Bakery HH Allam G . The potential pathogenic role of IL-17/Th17 cells in both type 1 and type 2 diabetes mellitus. BioMed Pharmacother. (2018) 101:287–92. doi: 10.1016/j.biopha.2018.02.103, PMID: 29499402

[17] Okeke EB Okwor I Uzonna JE . Regulatory T cells restrain CD4+ T cells from causing unregulated immune activation and hypersensitivity to lipopolysaccharide challenge. J Immunol. (2014) 193:655–62. doi: 10.4049/jimmunol.1303064, PMID: 24943218

[18] Zi C He L Yao H Ren Y He T Gao Y . Changes of Th17 cells, regulatory T cells, Treg/Th17, IL-17 and IL-10 in patients with type 2 diabetes mellitus: a systematic review and meta-analysis. Endocrine. (2022) 76:263–72. doi: 10.1007/s12020-022-03043-6, PMID: 35397088

[19] Kim JH Park K Lee SB Kang S Park JS Ahn CW . Relationship between natural killer cell activity and glucose control in patients with type 2 diabetes and prediabetes. J Diabetes Investig. (2019) 10:1223–8. doi: 10.1111/jdi.13002, PMID: 30618112 PMC6717814

[20] Mxinwa V Dludla PV Nyambuya TM Mokgalaboni K Mazibuko-Mbeje SE Nkambule BB . Natural killer cell levels in adults living with type 2 diabetes: a systematic review and meta-analysis of clinical studies. BMC Immunol. (2020) 21:51. doi: 10.1186/s12865-020-00378-5, PMID: 32907543 PMC7487809

[21] Wang H Cao K Liu S Xu Y Tang L . Tim-3 expression causes NK cell dysfunction in type 2 diabetes patients. Front Immunol. (2022) 13:852436. doi: 10.3389/fimmu.2022.852436, PMID: 35464400 PMC9018664

[22] Allahyani M Alshalawi AM Alshalawii MR Alqorashi SA Aljuaid A Almehmadi MM . Phenotypical evaluation of lymphocytes and monocytes in patients with type 2 diabetes mellitus in Saudi Arabia. Saudi Med J. (2023) 44:296–305. doi: 10.15537/smj.2023.44.3.20220873, PMID: 36940958 PMC10043885

[23] Cheng Y Yue L Wang Z Zhang J Xiang G . Hyperglycemia associated with lymphopenia and disease severity of COVID-19 in type 2 diabetes mellitus. J Diabetes Complicat. (2021) 35:107809. doi: 10.1016/j.jdiacomp.2020.107809, PMID: 33288414 PMC7690319

[24] Chen HL Wu C Cao L Wang R Zhang TY He Z . The association between the neutrophil-to-lymphocyte ratio and type 2 diabetes mellitus: a cross-sectional study. BMC Endocr Disord. (2024) 24:107. doi: 10.1186/s12902-024-01637-x, PMID: 38982402 PMC11232124

[25] Chen G Tan C Liu X Chen Y . Association between the neutrophil-to-lymphocyte ratio and diabetes secondary to exocrine pancreatic disorders. Front Endocrinol (Lausanne). (2022) 13:957129. doi: 10.3389/fendo.2022.957129, PMID: 35937787 PMC9352859

[26] Li H Chen M Wang Y Cui W Lou Y Chen D . The predictive value of tyG index and NLR for risk of CHD and the severity of coronary artery lesions in patients with type 2 diabetes mellitus. J Inflammation Res. (2024) 17:11813–28. doi: 10.2147/JIR.S496419, PMID: 39749002 PMC11694022

[27] Li J Wang X Jia W Wang K Wang W Diao W . Association of the systemic immuno-inflammation index, neutrophil-to-lymphocyte ratio, and platelet-to-lymphocyte ratio with diabetic microvascular complications. Front Endocrinol (Lausanne). (2024) 15:1367376. doi: 10.3389/fendo.2024.1367376, PMID: 38660516 PMC11039910

[28] Zhu B Liu Y Liu W Cao C Chen Y Yi Y . Association of neutrophil-to-lymphocyte ratio with all-cause and cardiovascular mortality in CVD patients with diabetes or pre-diabetes. Sci Rep. (2024) 14:24324. doi: 10.1038/s41598-024-74642-8, PMID: 39414853 PMC11484937

[29] Li D Zhong J Zhang Q Zhang J . Effects of anti-inflammatory therapies on glycemic control in type 2 diabetes mellitus. Front Immunol. (2023) 14:1125116. doi: 10.3389/fimmu.2023.1125116, PMID: 36936906 PMC10014557

[30] Widowati W Darsono L Utomo HS Sabrina AHN Natariza MR Valentinus Tarigan AC . Antidiabetic and hepatoprotection effect of butterfly pea flower (Clitoria ternatea L.) through antioxidant, anti-inflammatory, lower LDH, ACP, AST, and ALT on diabetes mellitus and dyslipidemia rat. Heliyon. (2024) 10:e29812. doi: 10.1016/j.heliyon.2024.e29812, PMID: 38681657 PMC11053275

[31] Nematollahi S Pishdad GR Zakerkish M Namjoyan F Ahmadi Angali K Borazjani F . The effect of berberine and fenugreek seed co-supplementation on inflammatory factor, lipid and glycemic profile in patients with type 2 diabetes mellitus: a double-blind controlled randomized clinical trial. Diabetol Metab Syndr. (2022) 14:120. doi: 10.1186/s13098-022-00888-9, PMID: 35999562 PMC9395822

[32] Ki M Baek S Yun YD Kim N Hyde M Na B . Age-related differences in diabetes care outcomes in Korea: a retrospective cohort study. BMC Geriatr. (2014) 14:111. doi: 10.1186/1471-2318-14-111, PMID: 25319086 PMC4210558

[33] Zhu M Liu X Liu W Lu Y Cheng J Chen Y . β cell aging and age-related diabetes. Aging (Albany NY). (2021) 13:7691–706. doi: 10.18632/aging.202593, PMID: 33686020 PMC7993693

[34] Odawara M Kadowaki T Naito Y . Incidence and predictors of hypoglycemia in Japanese patients with type 2 diabetes treated by insulin glargine and oral antidiabetic drugs in real-life: ALOHA post-marketing surveillance study sub-analysis. Diabetol Metab Syndr. (2014) 6:20. doi: 10.1186/1758-5996-6-20, PMID: 24528773 PMC3931675

[35] Bonadonna RC Mauricio D Müller-Wieland D Freemantle N Bigot G Mauquoi C . Impact of age on the effectiveness and safety of insulin glargine 300 U/mL: results from the REALI european pooled data analysis. Diabetes Ther. (2021) 12:1073–97. doi: 10.1007/s13300-021-01030-0, PMID: 33650085 PMC7994463

[36] Kautzky-Willer A Kosi L Lin J Mihaljevic R . Gender-based differences in glycaemic control and hypoglycaemia prevalence in patients with type 2 diabetes: results from patient-level pooled data of six randomized controlled trials. Diabetes Obes Metab. (2015) 17:533–40. doi: 10.1007/s13300-021-01030-0, PMID: 25678212 PMC6680342

[37] Owen V Seetho I Idris I . Predictors of responders to insulin therapy at 1 year among adults with type 2 diabetes. Diabetes Obes Metab. (2010) 12:865–70. doi: 10.1111/j.1463-1326.2010.01239.x, PMID: 20920038

[38] Sheng X Li T Hu Y Xiong CS Hu L . Correlation between blood glucose indexes generated by the flash glucose monitoring system and diabetic vascular complications. Diabetes Metab Syndr Obes. (2023) 16:2447–56. doi: 10.2147/DMSO.S418224, PMID: 37608851 PMC10440601

[39] Tuttle KR Bakris GL Bilous RW Chiang JL de Boer IH Goldstein-Fuchs J . Diabetic kidney disease: a report from an ADA Consensus Conference. Diabetes Care. (2014) 37:2864–83. doi: 10.2337/dc14-1296, PMID: 25249672 PMC4170131

[40] Whyte MB Joy M Hinton W McGovern A Hoang U van Vlymen J . Early and ongoing stable glycaemic control is associated with a reduction in major adverse cardiovascular events in people with type 2 diabetes: A primary care cohort study. Diabetes Obes Metab. (2022) 24:1310–8. doi: 10.1111/dom.14705, PMID: 35373891 PMC9320871

[41] Su G Mi SH Tao H Li Z Yang HX Zheng H . Impact of admission glycemic variability, glucose, and glycosylated hemoglobin on major adverse cardiac events after acute myocardial infarction. Diabetes Care. (2013) 36:1026–32. doi: 10.2337/dc12-0925, PMID: 23349547 PMC3609497

