# Supplementary material for: Risk factor screening and predictive modeling of time-in-range in patients with T2DM undergoing SIIT therapy
Source: Front Endocrinol (Lausanne). 2025 Dec 4;16:1664366. doi: 10.3389/fendo.2025.1664366 (PMC12711546; doi:10.3389/fendo.2025.1664366)

Supplementary Material

Supplementary Table1:Comparison of baseline clinical characteristics and laboratory parameters between the two cohorts

| Variable | Levels | Overall | Cohorts 1 | Cohorts 2 |
| --- | --- | --- | --- | --- |
|  |  | N = 796 | N = 145 | N = 651 |
| TCM |  |  |  |  |
|  | with | 458 ( 57.54%) | 75 ( 51.72%) | 383 ( 58.83%) |
|  | without | 338 ( 42.46%) | 70 ( 48.28%) | 268 ( 41.17%) |
| TCM Intake Day |  | 0 ( 0.00 - 7.00) | 0 ( 0.00 - 7.00) | 0 ( 0.00 - 7.00) |
| OADs |  |  |  |  |
|  | with | 28 ( 3.52%) | 7 ( 4.83%) | 21 ( 3.23%) |
|  | without | 768 ( 96.48%) | 138 ( 95.17%) | 630 ( 96.77%) |
| Metformin |  |  |  |  |
|  | with | 139 ( 17.46%) | 33 ( 22.76%) | 106 ( 16.28%) |
|  | without | 657 ( 82.54%) | 112 ( 77.24%) | 545 ( 83.72%) |
| MetDPP-4i |  |  |  |  |
|  | with | 783 ( 98.37%) | 145 ( 100.00%) | 638 ( 98.00%) |
|  | without | 13 ( 1.63%) | 0 ( 0.00%) | 13 ( 2.00%) |
| MetSGLT-2i |  |  |  |  |
|  | with | 785 ( 98.62%) | 143 ( 98.62%) | 642 ( 98.62%) |
|  | without | 11 ( 1.38%) | 2 ( 1.38%) | 9 ( 1.38%) |
| MetTZDs |  |  |  |  |
|  | with | 769 ( 96.61%) | 141 ( 97.24%) | 628 ( 96.47%) |
|  | without | 27 ( 3.39%) | 4 ( 2.76%) | 23 ( 3.53%) |
| DPP-4i |  |  |  |  |
|  | with | 415 ( 52.14%) | 66 ( 45.52%) | 349 ( 53.61%) |
|  | without | 381 ( 47.86%) | 79 ( 54.48%) | 302 ( 46.39%) |
| SGLT-2i |  |  |  |  |
|  | with | 711 ( 89.32%) | 135 ( 93.10%) | 576 ( 88.48%) |
|  | without | 85 ( 10.68%) | 10 ( 6.90%) | 75 ( 11.52%) |
| TZDs |  |  |  |  |
|  | with | 787 ( 98.87%) | 144 ( 99.31%) | 643 ( 98.77%) |
|  | without | 9 ( 1.13%) | 1 ( 0.69%) | 8 ( 1.23%) |
| Glinides |  |  |  |  |
|  | with | 711 ( 89.32%) | 120 ( 82.76%) | 591 ( 90.78%) |
|  | without | 85 ( 10.68%) | 25 ( 17.24%) | 60 ( 9.22%) |
| Sulfonylureas |  |  |  |  |
|  | with | 769 ( 96.61%) | 139 ( 95.86%) | 630 ( 96.77%) |
|  | without | 27 ( 3.39%) | 6 ( 4.14%) | 21 ( 3.23%) |
| a-Glucosidase inhibitor |  |  |  |  |
|  | with | 499 ( 62.69%) | 89 ( 61.38%) | 410 ( 62.98%) |
|  | without | 297 ( 37.31%) | 56 ( 38.62%) | 241 ( 37.02%) |
| GLP-1RA |  |  |  |  |
|  | with | 661 ( 83.04%) | 129 ( 88.97%) | 532 ( 81.72%) |
|  | without | 135 ( 16.96%) | 16 ( 11.03%) | 119 ( 18.28%) |
| T2DM Chronic complications |  |  |  |  |
|  | with | 233 ( 29.27%) | 44 ( 30.34%) | 189 ( 29.03%) |
|  | without | 563 ( 70.73%) | 101 ( 69.66%) | 462 ( 70.97%) |
| DR |  |  |  |  |
|  | with | 723 ( 90.83%) | 132 ( 91.03%) | 591 ( 90.78%) |
|  | without | 73 ( 9.17%) | 13 ( 8.97%) | 60 ( 9.22%) |
| DN |  |  |  |  |
|  | with | 444 ( 55.78%) | 75 ( 51.72%) | 369 ( 56.68%) |
|  | without | 352 ( 44.22%) | 70 ( 48.28%) | 282 ( 43.32%) |
| PAD |  |  |  |  |
|  | with | 455 ( 57.16%) | 80 ( 55.17%) | 375 ( 57.60%) |
|  | without | 341 ( 42.84%) | 65 ( 44.83%) | 276 ( 42.40%) |
| Diabetic Foot |  |  |  |  |
|  | with | 777 ( 97.61%) | 139 ( 95.86%) | 638 ( 98.00%) |
|  | without | 19 ( 2.39%) | 6 ( 4.14%) | 13 ( 2.00%) |
| Peripheral circulation complications |  |  |  |  |
|  | with | 742 ( 93.22%) | 131 ( 90.34%) | 611 ( 93.86%) |
|  | without | 54 ( 6.78%) | 14 ( 9.66%) | 40 ( 6.14%) |
| DKD |  |  |  |  |
|  | with | 547 ( 68.72%) | 105 ( 72.41%) | 442 ( 67.90%) |
|  | without | 249 ( 31.28%) | 40 ( 27.59%) | 209 ( 32.10%) |
| Metabolic Syndrome |  |  |  |  |
|  | with | 712 ( 89.45%) | 139 ( 95.86%) | 573 ( 88.02%) |
|  | without | 84 ( 10.55%) | 6 ( 4.14%) | 78 ( 11.98%) |
| Obesity |  |  |  |  |
|  | with | 689 ( 86.56%) | 136 ( 93.79%) | 553 ( 84.95%) |
|  | without | 107 ( 13.44%) | 9 ( 6.21%) | 98 ( 15.05%) |
| Fatty Liver |  |  |  |  |
|  | with | 470 ( 59.05%) | 87 ( 60.00%) | 383 ( 58.83%) |
|  | without | 326 ( 40.95%) | 58 ( 40.00%) | 268 ( 41.17%) |
| Hyperlipidemia |  |  |  |  |
|  | with | 587 ( 73.74%) | 117 ( 80.69%) | 470 ( 72.20%) |
|  | without | 209 ( 26.26%) | 28 ( 19.31%) | 181 ( 27.80%) |
| NASH |  |  |  |  |
|  | with | 769 ( 96.61%) | 143 ( 98.62%) | 626 ( 96.16%) |
|  | without | 27 ( 3.39%) | 2 ( 1.38%) | 25 ( 3.84%) |
| Hyperthyroidism |  |  |  |  |
|  | with | 787 ( 98.87%) | 142 ( 97.93%) | 645 ( 99.08%) |
|  | without | 9 ( 1.13%) | 3 ( 2.07%) | 6 ( 0.92%) |
| Hypothyroidism |  |  |  |  |
|  | with | 765 ( 96.11%) | 139 ( 95.86%) | 626 ( 96.16%) |
|  | without | 31 ( 3.89%) | 6 ( 4.14%) | 25 ( 3.84%) |
| Thyroid nodule |  |  |  |  |
|  | with | 509 ( 63.94%) | 99 ( 68.28%) | 410 ( 62.98%) |
|  | without | 287 ( 36.06%) | 46 ( 31.72%) | 241 ( 37.02%) |
| Thyroiditis |  |  |  |  |
|  | with | 787 ( 98.87%) | 145 ( 100.00%) | 642 ( 98.62%) |
|  | without | 9 ( 1.13%) | 0 ( 0.00%) | 9 ( 1.38%) |
| MACE |  |  |  |  |
|  | with | 616 ( 77.39%) | 102 ( 70.34%) | 514 ( 78.96%) |
|  | without | 180 ( 22.61%) | 43 ( 29.66%) | 137 ( 21.04%) |
| Hypertension |  |  |  |  |
|  | with | 330 ( 41.46%) | 58 ( 40.00%) | 272 ( 41.78%) |
|  | without | 466 ( 58.54%) | 87 ( 60.00%) | 379 ( 58.22%) |

Supplementary Table2: Baseline clinical features and laboratory parameters of patients in subgroups were compared

| Variable | Levels | Subgroup 1 | Subgroup 2 |
| --- | --- | --- | --- |
|  |  | N = 129 | N = 522 |
| TCM |  |  |  |
|  | with | 71 (55.04%) | 312 (59.77%) |
|  | without | 58 (44.96%) | 210 (40.23%) |
| TCM Intake Day |  | 0.00 (0.00 - 7.00) | 0.00 (0.00 - 7.00) |
| OADs |  |  |  |
|  | with | 1 (0.78%) | 20 (3.83%) |
|  | without | 128 (99.22%) | 502 (96.17%) |
| Metformin |  |  |  |
|  | with | 22 (17.05%) | 84 (16.09%) |
|  | without | 107 (82.95%) | 438 (83.91%) |
| MetDPP-4i |  |  |  |
|  | with | 126 (97.67%) | 512 (98.08%) |
|  | without | 3 (2.33%) | 10 (1.92%) |
| MetSGLT-2i |  |  |  |
|  | with | 128 (99.22%) | 514 (98.47%) |
|  | without | 1 (0.78%) | 8 (1.53%) |
| MetTZDs |  |  |  |
|  | with | 124 (96.12%) | 504 (96.55%) |
|  | without | 5 (3.88%) | 18 (3.45%) |
| DPP-4i |  |  |  |
|  | with | 73 (56.59%) | 276 (52.87%) |
|  | without | 56 (43.41%) | 246 (47.13%) |
| SGLT-2i |  |  |  |
|  | with | 115 (89.15%) | 461 (88.31%) |
|  | without | 14 (10.85%) | 61 (11.69%) |
| TZDs |  |  |  |
|  | with | 126 (97.67%) | 517 (99.04%) |
|  | without | 3 (2.33%) | 5 (0.96%) |
| Glinides |  |  |  |
|  | with | 112 (86.82%) | 479 (91.76%) |
|  | without | 17 (13.18%) | 43 (8.24%) |
| Sulfonylureas |  |  |  |
|  | with | 126 (97.67%) | 504 (96.55%) |
|  | without | 3 (2.33%) | 18 (3.45%) |
| a-Glucosidase inhibitor |  |  |  |
|  | with | 83 (64.34%) | 327 (62.64%) |
|  | without | 46 (35.66%) | 195 (37.36%) |
| GLP-1RA |  |  |  |
|  | with | 102 (79.07%) | 430 (82.38%) |
|  | without | 27 (20.93%) | 92 (17.62%) |
| T2DM Chronic complications |  |  |  |
|  | with | 30 (23.26%) | 159 (30.46%) |
|  | without | 99 (76.74%) | 363 (69.54%) |
| DR |  |  |  |
|  | with | 117 (90.70%) | 474 (90.80%) |
|  | without | 12 (9.30%) | 48 (9.20%) |
| DN |  |  |  |
|  | with | 58 (44.96%) | 311 (59.58%) |
|  | without | 71 (55.04%) | 211 (40.42%) |
| PAD |  |  |  |
|  | with | 73 (56.59%) | 302 (57.85%) |
|  | without | 56 (43.41%) | 220 (42.15%) |
| Diabetic Foot |  |  |  |
|  | with | 123 (95.35%) | 515 (98.66%) |
|  | without | 6 (4.65%) | 7 (1.34%) |
| Peripheral circulation complications |  |  |  |
|  | with | 116 (89.92%) | 495 (94.83%) |
|  | without | 13 (10.08%) | 27 (5.17%) |
| DKD |  |  |  |
|  | with | 86 (66.67%) | 356 (68.20%) |
|  | without | 43 (33.33%) | 166 (31.80%) |
| Metabolic Syndrome |  |  |  |
|  | with | 117 (90.70%) | 456 (87.36%) |
|  | without | 12 (9.30%) | 66 (12.64%) |
| Obesity |  |  |  |
|  | with | 106 (82.17%) | 447 (85.63%) |
|  | without | 23 (17.83%) | 75 (14.37%) |
| Fatty Liver |  |  |  |
|  | with | 82 (63.57%) | 301 (57.66%) |
|  | without | 47 (36.43%) | 221 (42.34%) |
| Hyperlipidemia |  |  |  |
|  | with | 102 (79.07%) | 368 (70.50%) |
|  | without | 27 (20.93%) | 154 (29.50%) |
| NASH |  |  |  |
|  | with | 123 (95.35%) | 503 (96.36%) |
|  | without | 6 (4.65%) | 19 (3.64%) |
| Hyperthyroidism |  |  |  |
|  | with | 126 (97.67%) | 519 (99.43%) |
|  | without | 3 (2.33%) | 3 (0.57%) |
| Hypothyroidism |  |  |  |
|  | with | 125 (96.90%) | 501 (95.98%) |
|  | without | 4 (3.10%) | 21 (4.02%) |
| Thyroid nodule |  |  |  |
|  | with | 79 (61.24%) | 331 (63.41%) |
|  | without | 50 (38.76%) | 191 (36.59%) |
| Thyroiditis |  |  |  |
|  | with | 126 (97.67%) | 516 (98.85%) |
|  | without | 3 (2.33%) | 6 (1.15%) |
| MACE |  |  |  |
|  | with | 92 (71.32%) | 422 (80.84%) |
|  | without | 37 (28.68%) | 100 (19.16%) |
| Hypertension |  |  |  |
|  | with | 56 (43.41%) | 216 (41.38%) |
|  | without | 73 (56.59%) | 306 (58.62%) |

Supplementary Table3: Logistic regression analysis of factors associated with TBR events during hospitalization

| Variables | β | S.E | Z | *P* | OR (95%CI) |
| --- | --- | --- | --- | --- | --- |
|  |  |  |  |  |  |
| Intercept | -0.19 | 0.76 | -0.26 | 0.797 | 0.82 (0.19 ~ 3.63) |
| Length of hospital stay | 0.17 | 0.03 | 6.62 | **<.001** | 1.18 (1.12 ~ 1.24) |
| Weight | -0.03 | 0.01 | -3.18 | **0.001** | 0.97 (0.95 ~ 0.99) |
| Lymphocyte | -0.25 | 0.18 | -1.43 | 0.153 | 0.78 (0.55 ~ 1.10) |
| FCP（空） | -1.22 | 0.34 | -3.55 | **<.001** | 0.30 (0.15 ~ 0.58) |
| SGLT-2i |  |  |  |  |  |
| 0 |  |  |  |  | 1.00 (Reference) |
| 1 | -0.94 | 0.55 | -1.71 | 0.088 | 0.39 (0.13 ~ 1.15) |
| Hypothyroidism |  |  |  |  |  |
| 0 |  |  |  |  | 1.00 (Reference) |
| 1 | 1.07 | 0.46 | 2.35 | **0.019** | 2.92 (1.19 ~ 7.16) |
| Thyroid nodule |  |  |  |  |  |
| 0 |  |  |  |  | 1.00 (Reference) |
| 1 | -0.48 | 0.24 | -2.01 | **0.044** | 0.62 (0.39 ~ 0.99) |
| Hypertension |  |  |  |  |  |
| 0 |  |  |  |  | 1.00 (Reference) |
| 1 | -0.51 | 0.22 | -2.30 | **0.021** | 0.60 (0.39 ~ 0.93) |
| OR: Odds Ratio, CI: Confidence Interval | | | | | |

Supplementary Table4: Baseline characteristics of patients grouped by glinide use

|  |  | N = 796 | N = 711 | N = 85 |  |
| --- | --- | --- | --- | --- | --- |
| Variable | Levels | Overall | No glinide use | Glinide use | p-value |
| HbA1c |  | 9.30 (8.00 - 10.75) | 9.30 (7.90 - 10.70) | 9.40 (8.10 - 11.10) | 0.51 |
| FBG |  | 8.51 (6.46 - 10.89) | 8.40 (6.40 - 10.86) | 9.10 (7.37 - 10.93) | 0.12 |
| Age |  | 64.00 (54.00 - 73.00) | 64.00 (54.00 - 72.00) | 66.00 (60.00 - 77.00) | 0.002 |
| Length_of_hospital_stay |  | 12.00 (10.00 - 15.00) | 12.00 (10.00 - 15.00) | 14.00 (10.00 - 17.00) | 0.023 |
| High |  | 1.65 (1.58 - 1.70) | 1.65 (1.58 - 1.70) | 1.64 (1.57 - 1.70) | 0.369 |
| Weight |  | 67.00 (59.00 - 75.00) | 67.00 (59.50 - 75.00) | 63.00 (56.00 - 75.00) | 0.157 |
| BMI |  | 24.80 (22.31 - 26.99) | 24.86 (22.41 - 26.99) | 24.12 (21.30 - 26.99) | 0.282 |
| TG |  | 1.45 (1.03 - 2.13) | 1.44 (1.03 - 2.11) | 1.58 (0.97 - 2.15) | 0.817 |
| TyG |  | 9.19 (8.68 - 9.72) | 9.19 (8.68 - 9.70) | 9.16 (8.71 - 9.86) | 0.634 |
| TC |  | 0.96 (0.82 - 1.17) | 0.96 (0.82 - 1.17) | 1.01 (0.87 - 1.14) | 0.257 |
| LDL |  | 2.48 (1.91 - 3.10) | 2.49 (1.91 - 3.10) | 2.32 (1.86 - 3.09) | 0.458 |
| HDL |  | 0.96 (0.82 - 1.17) | 0.96 (0.82 - 1.17) | 1.01 (0.87 - 1.14) | 0.257 |
| Neutrophils |  | 3.70 (2.90 - 4.85) | 3.70 (2.90 - 4.80) | 3.50 (2.90 - 5.00) | 0.867 |
| Lymphocyte |  | 1.80 (1.40 - 2.30) | 1.80 (1.40 - 2.30) | 1.70 (1.30 - 2.10) | 0.083 |
| NLR |  | 2.06 (1.53 - 2.90) | 2.05 (1.50 - 2.89) | 2.13 (1.61 - 2.92) | 0.196 |
| FINS |  | 8.10 (4.90 - 12.70) | 8.20 (4.90 - 13.10) | 7.40 (5.20 - 11.20) | 0.553 |
| FCP |  | 0.68 (0.49 - 0.96) | 0.68 (0.49 - 0.96) | 0.69 (0.54 - 0.96) | 0.532 |
| Fist_Day_with_TIR |  | 5.00 (2.00 - 8.00) | 5.00 (2.00 - 8.00) | 6.00 (3.00 - 9.00) | 0.256 |
| Fist_Day_with_TIR_TITR |  | 6.00 (2.00 - 9.00) | 6.00 (2.00 - 9.00) | 6.00 (0.00 - 9.00) | 0.611 |
| Fist_Day_with_low_MAGE |  | 4.00 (2.00 - 6.00) | 4.00 (2.00 - 6.00) | 4.00 (2.00 - 6.00) | 0.83 |
| TBR_muns |  | 0.00 (0.00 - 0.00) | 0.00 (0.00 - 0.00) | 0.00 (0.00 - 0.00) | 0.576 |
| TBR |  |  |  |  | 0.602 |
|  | 0 | 671.00 (84.30%) | 601.00 (84.53%) | 70.00 (82.35%) |  |
|  | 1 | 125.00 (15.70%) | 110.00 (15.47%) | 15.00 (17.65%) |  |
| Sex |  |  |  |  | 0.737 |
|  | 0 | 463.00 (58.17%) | 415.00 (58.37%) | 48.00 (56.47%) |  |
|  | 1 | 333.00 (41.83%) | 296.00 (41.63%) | 37.00 (43.53%) |  |
| TCM |  |  |  |  | 0.157 |
|  | 0 | 458.00 (57.54%) | 403.00 (56.68%) | 55.00 (64.71%) |  |
|  | 1 | 338.00 (42.46%) | 308.00 (43.32%) | 30.00 (35.29%) |  |
| Metformin |  |  |  |  | 0.962 |
|  | 0 | 139.00 (17.46%) | 124.00 (17.44%) | 15.00 (17.65%) |  |
|  | 1 | 657.00 (82.54%) | 587.00 (82.56%) | 70.00 (82.35%) |  |
| MetDPP_4i |  |  |  |  | 0.725 |
|  | 0 | 783.00 (98.37%) | 699.00 (98.31%) | 84.00 (98.82%) |  |
|  | 1 | 13.00 (1.63%) | 12.00 (1.69%) | 1.00 (1.18%) |  |
| MetSGLT_2i |  |  |  |  | 0.248 |
|  | 0 | 785.00 (98.62%) | 700.00 (98.45%) | 85.00 (100.00%) |  |
|  | 1 | 11.00 (1.38%) | 11.00 (1.55%) | 0.00 (0.00%) |  |
| MetTZD |  |  |  |  | 0.233 |
|  | 0 | 769.00 (96.61%) | 685.00 (96.34%) | 84.00 (98.82%) |  |
|  | 1 | 27.00 (3.39%) | 26.00 (3.66%) | 1.00 (1.18%) |  |
| DPP_4i |  |  |  |  | 0.056 |
|  | 0 | 415.00 (52.14%) | 379.00 (53.31%) | 36.00 (42.35%) |  |
|  | 1 | 381.00 (47.86%) | 332.00 (46.69%) | 49.00 (57.65%) |  |
| SGLT_2i |  |  |  |  | 0.13 |
|  | 0 | 711.00 (89.32%) | 631.00 (88.75%) | 80.00 (94.12%) |  |
|  | 1 | 85.00 (10.68%) | 80.00 (11.25%) | 5.00 (5.88%) |  |
| TZD |  |  |  |  | 0.297 |
|  | 0 | 787.00 (98.87%) | 702.00 (98.73%) | 85.00 (100.00%) |  |
|  | 1 | 9.00 (1.13%) | 9.00 (1.27%) | 0.00 (0.00%) |  |
| Sulfonylureas |  |  |  |  | 0.479 |
|  | 0 | 769.00 (96.61%) | 688.00 (96.77%) | 81.00 (95.29%) |  |
|  | 1 | 27.00 (3.39%) | 23.00 (3.23%) | 4.00 (4.71%) |  |
| a__Glucosidase_inhibitor |  |  |  |  | 0.309 |
|  | 0 | 499.00 (62.69%) | 450.00 (63.29%) | 49.00 (57.65%) |  |
|  | 1 | 297.00 (37.31%) | 261.00 (36.71%) | 36.00 (42.35%) |  |
| GLP_1RA |  |  |  |  | 0.665 |
|  | 0 | 661.00 (83.04%) | 589.00 (82.84%) | 72.00 (84.71%) |  |
|  | 1 | 135.00 (16.96%) | 122.00 (17.16%) | 13.00 (15.29%) |  |
| Ultra_short_acting_insulin |  |  |  |  | 0.034 |
|  | 0 | 299.00 (37.56%) | 276.00 (38.82%) | 23.00 (27.06%) |  |
|  | 1 | 497.00 (62.44%) | 435.00 (61.18%) | 62.00 (72.94%) |  |
| Short_acting_insulin |  |  |  |  | 0.001 |
|  | 0 | 630.00 (79.15%) | 574.00 (80.73%) | 56.00 (65.88%) |  |
|  | 1 | 166.00 (20.85%) | 137.00 (19.27%) | 29.00 (34.12%) |  |
| Isohpane_Insulin |  |  |  |  | <0.001 |
|  | 0 | 761.00 (95.60%) | 694.00 (97.61%) | 67.00 (78.82%) |  |
|  | 1 | 35.00 (4.40%) | 17.00 (2.39%) | 18.00 (21.18%) |  |
| Long_acting_insulin |  |  |  |  | <0.001 |
|  | 0 | 668.00 (83.92%) | 631.00 (88.75%) | 37.00 (43.53%) |  |
|  | 1 | 128.00 (16.08%) | 80.00 (11.25%) | 48.00 (56.47%) |  |
| premixed_insulin |  |  |  |  | <0.001 |
|  | 0 | 168.00 (21.11%) | 119.00 (16.74%) | 49.00 (57.65%) |  |
|  | 1 | 628.00 (78.89%) | 592.00 (83.26%) | 36.00 (42.35%) |  |
| OADS_types |  |  |  |  | <0.001 |
|  | 0 | 28.00 (3.52%) | 28.00 (3.94%) | 0.00 (0.00%) |  |
|  | 1 | 197.00 (24.75%) | 195.00 (27.43%) | 2.00 (2.35%) |  |
|  | 2 | 370.00 (46.48%) | 346.00 (48.66%) | 24.00 (28.24%) |  |
|  | 3 | 155.00 (19.47%) | 116.00 (16.32%) | 39.00 (45.88%) |  |
|  | 4 | 41.00 (5.15%) | 24.00 (3.38%) | 17.00 (20.00%) |  |
|  | 5 | 4.00 (0.50%) | 2.00 (0.28%) | 2.00 (2.35%) |  |
|  | 6 | 1.00 (0.13%) | 0.00 (0.00%) | 1.00 (1.18%) |  |
| T2DM_Chronic_complications |  |  |  |  | 0.218 |
|  | 0 | 233.00 (29.27%) | 213.00 (29.96%) | 20.00 (23.53%) |  |
|  | 1 | 563.00 (70.73%) | 498.00 (70.04%) | 65.00 (76.47%) |  |
| DR |  |  |  |  | 0.266 |
|  | 0 | 723.00 (90.83%) | 643.00 (90.44%) | 80.00 (94.12%) |  |
|  | 1 | 73.00 (9.17%) | 68.00 (9.56%) | 5.00 (5.88%) |  |
| DN |  |  |  |  | 0.924 |
|  | 0 | 444.00 (55.78%) | 397.00 (55.84%) | 47.00 (55.29%) |  |
|  | 1 | 352.00 (44.22%) | 314.00 (44.16%) | 38.00 (44.71%) |  |
| PAD |  |  |  |  | 0.576 |
|  | 0 | 455.00 (57.16%) | 404.00 (56.82%) | 51.00 (60.00%) |  |
|  | 1 | 341.00 (42.84%) | 307.00 (43.18%) | 34.00 (40.00%) |  |
| Diabetic_Foot |  |  |  |  | 0.439 |
|  | 0 | 777.00 (97.61%) | 693.00 (97.47%) | 84.00 (98.82%) |  |
|  | 1 | 19.00 (2.39%) | 18.00 (2.53%) | 1.00 (1.18%) |  |
| Peripheral_circulation_complications |  |  |  |  | <0.001 |
|  | 0 | 742.00 (93.22%) | 674.00 (94.80%) | 68.00 (80.00%) |  |
|  | 1 | 54.00 (6.78%) | 37.00 (5.20%) | 17.00 (20.00%) |  |
| DKD |  |  |  |  | 0.551 |
|  | 0 | 547.00 (68.72%) | 491.00 (69.06%) | 56.00 (65.88%) |  |
|  | 1 | 249.00 (31.28%) | 220.00 (30.94%) | 29.00 (34.12%) |  |
| Metabolic_Syndrome |  |  |  |  | 0.462 |
|  | 0 | 712.00 (89.45%) | 634.00 (89.17%) | 78.00 (91.76%) |  |
|  | 1 | 84.00 (10.55%) | 77.00 (10.83%) | 7.00 (8.24%) |  |
| Obesity |  |  |  |  | 0.386 |
|  | 0 | 689.00 (86.56%) | 618.00 (86.92%) | 71.00 (83.53%) |  |
|  | 1 | 107.00 (13.44%) | 93.00 (13.08%) | 14.00 (16.47%) |  |
| Fatty_Liver |  |  |  |  | 0.672 |
|  | 0 | 470.00 (59.05%) | 418.00 (58.79%) | 52.00 (61.18%) |  |
|  | 1 | 326.00 (40.95%) | 293.00 (41.21%) | 33.00 (38.82%) |  |
| Hyperlipidemia |  |  |  |  | 0.545 |
|  | 0 | 587.00 (73.74%) | 522.00 (73.42%) | 65.00 (76.47%) |  |
|  | 1 | 209.00 (26.26%) | 189.00 (26.58%) | 20.00 (23.53%) |  |
| NASH |  |  |  |  | 0.233 |
|  | 0 | 769.00 (96.61%) | 685.00 (96.34%) | 84.00 (98.82%) |  |
|  | 1 | 27.00 (3.39%) | 26.00 (3.66%) | 1.00 (1.18%) |  |
| Hyperthyroidism |  |  |  |  | 0.966 |
|  | 0 | 787.00 (98.87%) | 703.00 (98.87%) | 84.00 (98.82%) |  |
|  | 1 | 9.00 (1.13%) | 8.00 (1.13%) | 1.00 (1.18%) |  |
| Hypothyroidism |  |  |  |  | 0.171 |
|  | 0 | 765.00 (96.11%) | 681.00 (95.78%) | 84.00 (98.82%) |  |
|  | 1 | 31.00 (3.89%) | 30.00 (4.22%) | 1.00 (1.18%) |  |
| Thyroid_nodule |  |  |  |  | 0.298 |
|  | 0 | 509.00 (63.94%) | 459.00 (64.56%) | 50.00 (58.82%) |  |
|  | 1 | 287.00 (36.06%) | 252.00 (35.44%) | 35.00 (41.18%) |  |
| Thyroiditis |  |  |  |  | 0.966 |
|  | 0 | 787.00 (98.87%) | 703.00 (98.87%) | 84.00 (98.82%) |  |
|  | 1 | 9.00 (1.13%) | 8.00 (1.13%) | 1.00 (1.18%) |  |
| MACE |  |  |  |  | 0.542 |
|  | 0 | 616.00 (77.39%) | 548.00 (77.07%) | 68.00 (80.00%) |  |
|  | 1 | 180.00 (22.61%) | 163.00 (22.93%) | 17.00 (20.00%) |  |
| Hypertension |  |  |  |  | 0.859 |
|  | 0 | 330.00 (41.46%) | 294.00 (41.35%) | 36.00 (42.35%) |  |
|  | 1 | 466.00 (58.54%) | 417.00 (58.65%) | 49.00 (57.65%) |  |
| TIR_in_10_Length |  |  |  |  | 0.005 |
|  | 0 | 145.00 (18.22%) | 120.00 (16.88%) | 25.00 (29.41%) |  |
|  | 1 | 651.00 (81.78%) | 591.00 (83.12%) | 60.00 (70.59%) |  |
| MAGE_in_10_Length |  |  |  |  | 0.007 |
|  | 0 | 76.00 (9.55%) | 61.00 (8.58%) | 15.00 (17.65%) |  |
|  | 1 | 720.00 (90.45%) | 650.00 (91.42%) | 70.00 (82.35%) |  |
| TBR_muns |  |  |  |  | 0.866 |
|  | 0 | 671.00 (84.30%) | 601.00 (84.53%) | 70.00 (82.35%) |  |
|  | 1 | 92.00 (11.56%) | 82.00 (11.53%) | 10.00 (11.76%) |  |
|  | 2 | 22.00 (2.76%) | 18.00 (2.53%) | 4.00 (4.71%) |  |
|  | 3 | 5.00 (0.63%) | 4.00 (0.56%) | 1.00 (1.18%) |  |
|  | 4 | 3.00 (0.38%) | 3.00 (0.42%) | 0.00 (0.00%) |  |
|  | 5 | 2.00 (0.25%) | 2.00 (0.28%) | 0.00 (0.00%) |  |
|  | 6 | 1.00 (0.13%) | 1.00 (0.14%) | 0.00 (0.00%) |  |
| Insulin_types |  |  |  |  | <0.001 |
|  | 1 | 262.00 (32.91%) | 243.00 (34.18%) | 19.00 (22.35%) |  |
|  | 2 | 428.00 (53.77%) | 392.00 (55.13%) | 36.00 (42.35%) |  |
|  | 3 | 90.00 (11.31%) | 70.00 (9.85%) | 20.00 (23.53%) |  |
|  | 4 | 14.00 (1.76%) | 6.00 (0.84%) | 8.00 (9.41%) |  |
|  | 5 | 2.00 (0.25%) | 0.00 (0.00%) | 2.00 (2.35%) |  |
| Insulin_pump |  |  |  |  | 0.064 |
|  | 0 | 268.00 (33.67%) | 247.00 (34.74%) | 21.00 (24.71%) |  |
|  | 1 | 528.00 (66.33%) | 464.00 (65.26%) | 64.00 (75.29%) |  |

Supplementary Fig. 1:Patient hospitalization


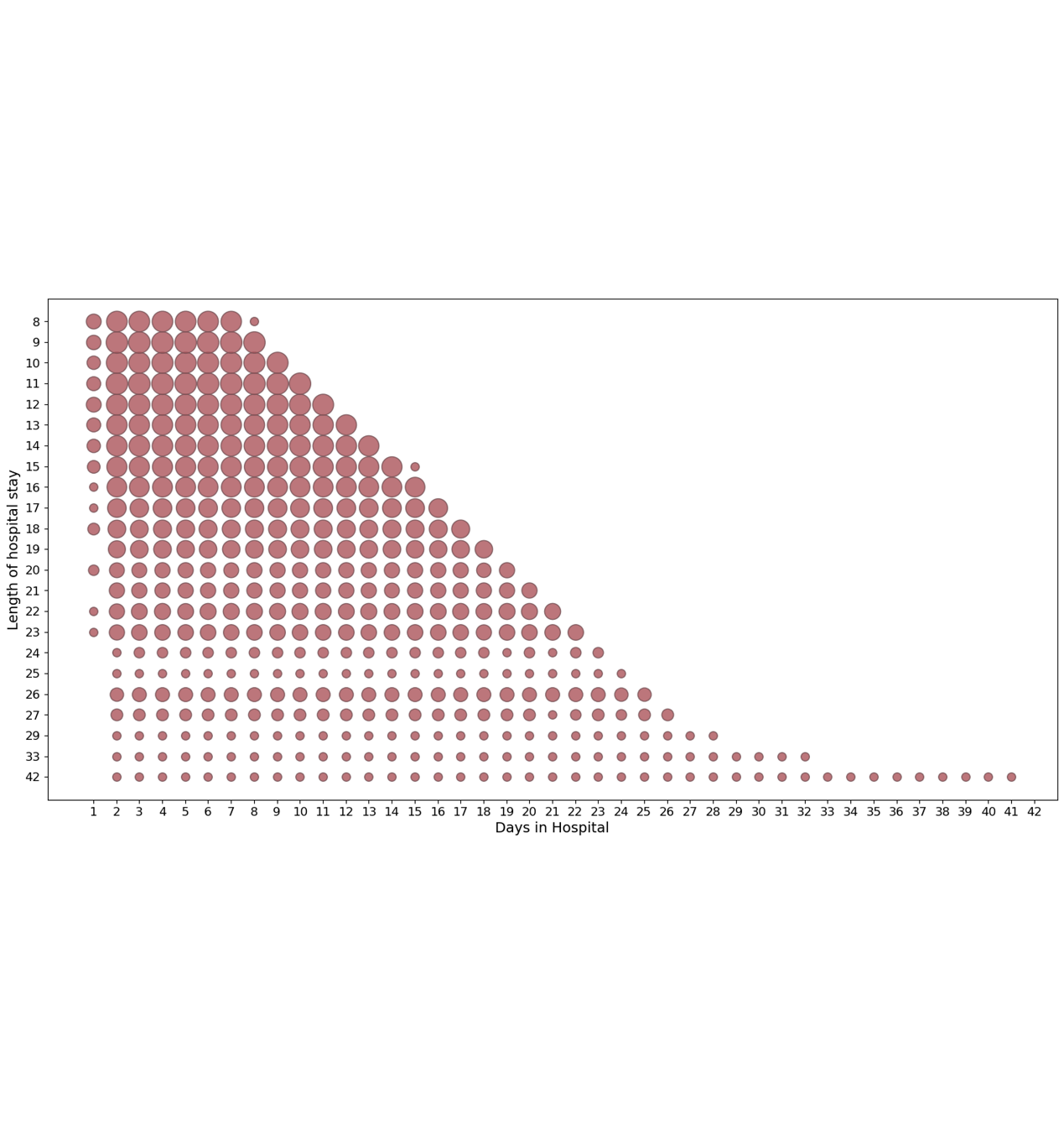

Supplement: Supplementary file 1 [file DataSheet1.docx]
